# Process development and preclinical evaluation of a major *Plasmodium falciparum* blood stage vaccine candidate, Cysteine-Rich Protective Antigen (CyRPA)

**DOI:** 10.3389/fimmu.2022.1005332

**Published:** 2022-09-22

**Authors:** Anjali Somanathan, Syed Yusuf Mian, Kritika Chaddha, Seemalata Uchoi, Praveen K. Bharti, Ravi Tandon, Deepak Gaur, Virander Singh Chauhan

**Affiliations:** ^1^ Laboratory of Malaria and Vaccine Research, School of Biotechnology, Jawaharlal Nehru University, New Delhi, India; ^2^ICMR-National Institute of Research in Tribal Health (NIRTH), Jabalpur, India; ^3^Laboratory of AIDS Research and Immunology, School of Biotechnology, Jawaharlal Nehru University, New Delhi, India; ^4^ Malaria Group, International Centre for Genetic Engineering and Biotechnology (ICGEB), New Delhi, India

**Keywords:** *Plasmodium falciparum*, CyRPA, gia, adjuvants, recombinant subunit malaria vaccines, humoral and cellular response, *E. coli*, fermentation

## Abstract

*Plasmodium falciparum* Cysteine-Rich Protective Antigen (CyRPA) is an essential, highly conserved merozoite antigen that forms an important multi-protein complex (RH5/Ripr/CyRPA) necessary for erythrocyte invasion. CyRPA is a promising blood-stage vaccine target that has been shown to elicit potent strain-transcending parasite neutralizing antibodies. Recently, we demonstrated that naturally acquired immune anti-CyRPA antibodies are invasion-inhibitory and therefore a correlate of protection against malaria. Here, we describe a process for the large-scale production of tag-free CyRPA vaccine in *E. coli* and demonstrate its parasite neutralizing efficacy with commonly used adjuvants. CyRPA was purified from inclusion bodies using a one-step purification method with high purity (>90%). Biochemical and biophysical characterization showed that the purified tag-free CyRPA interacted with RH5, readily detected by a conformation-specific CyRPA monoclonal antibody and recognized by sera from malaria infected individuals thus indicating that the recombinant antigen was correctly folded and retained its native conformation. Tag-free CyRPA formulated with Freund’s adjuvant elicited highly potent parasite neutralizing antibodies achieving inhibition of >90% across diverse parasite strains. Importantly, we identified tag-free CyRPA/Alhydrogel formulation as most effective in inducing a highly immunogenic antibody response that exhibited efficacious, cross-strain *in vitro* parasite neutralization achieving ~80% at 10 mg/ml. Further, CyRPA/Alhydrogel vaccine induced anti-parasite cytokine response in mice. In summary, our study provides a simple, scalable, cost-effective process for the production of tag-free CyRPA that in combination with human-compatible adjuvant induces efficacious humoral and cell-mediated immune response.

## Introduction

*P. falciparum* is a leading cause of malaria that annually accounts for ~200 million cases and ~0.6 million deaths (1). In the wake of rising resistance to insecticides and anti-malarials, development of an effective malaria vaccine is a major global health priority (2, 3). RTS,S/AS01 was recently recommended by WHO for widespread use among children in Africa and declared as the first malaria vaccine (4). However, the vaccine is poorly efficacious and lacks durability (5, 6). Therefore, extensive efforts need to continue to identify potent vaccine targets and develop novel delivery platforms that generate optimal protection and long-lasting memory response.

Blood-stage of the parasite’s life cycle, which is responsible for malaria pathogenesis and all associated clinical manifestations, is the primary target of naturally acquired immunity to malaria (7, 8). Vaccines targeting this stage therefore have the potential to elicit protective immune response, prevent malaria pathogenesis and reduce malaria transmission (9–11). Past efforts to develop efficacious blood-stage vaccines have failed due to high antigenic polymorphism and redundant erythrocyte invasion pathways of *P. falciparum* leading to strain-specific immune response and immune evasion by the parasite (12, 13). It is therefore crucial to identify conserved and essential parasite antigens that can elicit a potent, strain-transcending parasite neutralizing immune response.

Studies in the last decade have identified several key parasite antigens that have been shown to be essential for parasite survival, exhibit limited polymorphism and elicit potent parasite neutralizing antibodies *in vitro* and *in vivo (* 14, 15). One of these antigens is the Cysteine Rich Protective Antigen (CyRPA) that participates in the formation of an essential multiprotein complex, RH5/Ripr/CyRPA, on the merozoites surface necessary for successful erythrocyte invasion (16, 17). Initial studies demonstrated that CyRPA provides protection against *Plasmodium* infection in an NSG mouse model (18) and plays an important role in calcium mediated signalling during erythrocyte invasion (17, 19). Our lab showed that CyRPA is an essential component of the RH5 multiprotein complex and induces highly efficacious strain-transcending parasite neutralizing antibodies that synergizes with RH5 antibodies (16). Subsequent study from the lab identified a CyRPA based antibody combination, RH5+CyRPA+MSP-1_19_, as an efficacious multi-component vaccine that elicited robust antibody response against *P. falciparum* laboratory clones and clinical isolates (20). Other studies have shown that CyRPA based combinations with antibodies against other parasite antigens (EBA-181, MSRP-5, RAMA, Ripr) also exhibit synergistic invasion inhibition (21, 22). Naturally acquired antibodies targeting CyRPA have been shown to inhibit erythrocyte invasion and provide protection from malaria re-infection (23, 24). Furthermore, in a recent report, virosome-based CyRPA vaccine was shown to provide protection in *in vitro* and *in vivo* models (25). Taken together, these studies provide strong support for the development of CyRPA based malaria vaccine.

Despite the success in the identification of several important vaccine targets, one of the major challenges in developing a protein subunit vaccine against malaria has been the difficulty of producing the antigen recombinantly in their native, conformationally stable form that is immunogenic and able to induce high-titer broadly neutralizing antibodies (26). The recombinant production of the leading blood-stage vaccine candidate, RH5 has faced similar challenges (27–29). Similarly MSP-1_19_, which is another major blood-stage vaccine target, was poorly immunogenic in recombinant form and therefore, produced in fusion with an MSP-3 construct for enhancing its immunogenicity (30–32). CyRPA is among the leading vaccine candidates and a scalable process for its production in bacterial expression system is still lacking. Our lab has previously successfully demonstrated the production of recombinant functionally active CyRPA that exhibited high immunogenicity, induced neutralizing antibodies in small animals and recognizable by sera from malaria infected individuals (16, 23). However, all these studies were done at a shake flask level and involved expression of CyRPA with a hexa-histidine tag, the use of which in human vaccines is a safety concern in various countries including India. Besides, all these studies have been done with Freund’s adjuvant, which is human incompatible. To address these concerns and achieve the goal of developing a CyRPA based vaccine, we conducted the present study in which we successfully purify CyRPA without any tag (tag-free CyRPA) through a single-step purification method, defined a cost-effective process of its large-scale production and performed a comprehensive screening of several adjuvants to identify best CyRPA vaccine formulation for human use.

We demonstrated using various biochemical, biophysical and immunological assays that the recombinant tag-free CyRPA mimics the native form of the protein both in structure and function. Using the standard growth inhibitory assays, we have shown that the recombinant CyRPA is a target of potent, broadly neutralizing antibodies. Further, we have identified a CyRPA vaccine formulated with a human-compatible adjuvant that induces cross-strain humoral as well as cellular immunity. Collectively, this is the first report of a process for the large-scale production of a full-length recombinant CyRPA in *E. coli* that elicited a highly potent immunogenic response, thus would help for further preclinical and clinical evaluation of CyRPA in combination with other parasite antigens.

## Materials and methods

### Expression, fermentation and protein purification

The DNA fragment encoding the full-length CyRPA (R_31_-E_362_) of 3D7 *P. falciparum* clone was codon optimized (Gene Art; Life Technologies) and subcloned into pET24b upstream to a 6xHis tag encoding region (Novagen) as reported previously (16). To clone the tag-free CyRPA, a stop codon was inserted at the 3’end of the codon optimized CyRPA gene using specific primers. The insertion of the stop codon was confirmed by sequencing. The plasmid construct encoding the tag-free CyRPA was transformed into *E. coli* expression cell strains to screen for target protein expression in Luria Bertani (LB) broth media at shake flask level. Some *E.coli* strains co-expressed the GroEL-ES chaperone system which were used to check expression in supernatant fraction.

Before fermentation*, E. coli* BL21 (DE3) culture was adapted in a complex medium (select soytone 1.6% and yeast extract 1%), which has all components of non-animal origin, at shake flask level. Fermentation was carried out in a 7 L AppliKon Biotechnology B.V. bioreactor at 4 L, equipped with extensive analytical devices for real time measurement of pH, temperature and dissolved oxygen. The fermenter containing media components was sterilized by autoclaving at 121°C for 20 mins, after which filter-sterilized kanamycin (50 mg/L) was added to the cooled fermenter. Fermentation run was started by inoculating 2% (v/v) of overnight starter culture. Fermentation parameters were set and maintained as agitation ~1500 rpm, temperature ~37°C, pH 7.0 and DO at 30%. Research cell banks (RCBs) of positive BL21 (DE3) clones were also prepared in complex as well as LB media and stored at -80°C.

The biomass was resuspended using 10-fold cell lysis buffer (25 mM Tris, 300 mM NaCl, 100 mM NaH_2_PO_4_, 1% Glycerol, 5 mM Benzamidine HCl [pH 7.4]) and sonicated after which protein inclusion bodies (IBs) were collected by centrifugation at 21000g for 1 h. These IBs were subjected to washing thrice using endotoxin wash buffer (20 mM Tris, 500 mM NaCl, 2 mM β-Mercaptoethanol, 10 mM EDTA, 5%Triton X-114 [pH 7.4]), thrice with wash buffer 1 (20 mM Tris, 500 mM NaCl, 2 mM β-Mercaptoethanol, 10 mM EDTA, 1%Triton X100 [pH 7.4]) and twice with wash buffer 2 (20 mM Tris, 500 mM NaCl [pH 7.4]). The IBs were resuspended in solubilisation buffer (20 mM Tris, 50 mM Na_2_HPO_4_, 6 M Guanidine HCl, 300 mM NaCl, 2 mM β-mercaptoethanol [pH 7.4]) under denaturing conditions, homogenized and kept overnight with constant stirring at room temperature (RT). After clarification by centrifugation, the solubilized and denatured protein was subjected to refolding by diluting it 26-fold in pre-chilled Tris-based refolding buffer (55 mM Tris, 264 mM NaCl, 11 mM KCl, 2.2 mM MgCl_2_, 2.2 mM CaCl_2_, 440 mM Sucrose, 550 mM L-Arginine, 0.1 mM oxidised glutathione, 1 mM reduced glutathione [pH 8]) under redox conditions with constant stirring and kept overnight at 4˚C. Next day, the refolded sample was centrifuged at high speed (21000g) to remove any precipitated insoluble material following which the protein was put up for dialysis against 15 fold Tris-based buffer (25 mM Tris, 50 mM NaCl, 100 mM Urea [pH 8]) at 4˚C for ~20 hrs. The dialysis buffer was changed once after 6-8 hrs, after which the protein was collected and filtered to remove any precipitation.

The refolded and dialysed tag-free CyRPA was purified by anion exchange chromatography. Around 0.5 ml Q-Sepharose (Cytiva Life Sciences) resin per 1 mg of solubilised protein was packed in an XK-16 column (Cytiva Life Sciences), washed with Milli-Q and equilibrated with buffer A (25 mM Tris [pH 8.0]). The dialyzed protein was then loaded on the anion exchange column at the flow rate of 1 ml/min on AKTA (Explorer; Amersham Biosciences). The column was washed with 100 mM NaCl concentration and the bound protein was eluted in a step-wise gradient of NaCl (0.15 M, 0.25 M, 0.3 M, 0.4 M, 0.5 M and 1 M) using buffer B (25 mM Tris, 1 M NaCl [pH 8.0]). The elutes were collected at 0.15 M, 0.25 M, 0.3 M, 0.4 M, 0.5 M and 1 M in different fractions with the protein of interest observed only from 0.3 M to 1 M fractions by SDS-PAGE.

### Size exclusion chromatography

The ion-exchange (IEx) purified elutes collected at 0.3 M, 0.4 M, 0.5 M and 1 M were loaded individually on SEC column (Superdex 75 [16/600]; Cytiva Life Sciences) to identify their monomeric or oligomeric state. To identify the monomeric or oligomeric protein peaks, known molecular weights (Mw) protein markers (28-4038-42; Cytiva Life Sciences) were also run and used to plot the calibration curve (K_av_ versus log Mw) Using this calibration curve, molecular weight of unknown protein samples were determined. Accordingly, the 0.3 M elutes had a single peak corresponding to monomeric CyRPA, while 0.4 M, 0.5 M and 1 M CyRPA contained a mixture of monomer and high order oligomers.

### LC-MS analysis

Samples for LC-MS (liquid chromatography-mass spectrometry) analysis were prepared as described previously (33). Briefly, the two protein bands were excised from the gel followed by reduction with 5 mM TCEP, further alkylation with 50 mM iodoacetamide and then trypsin digestion for 16 h at 37°C. The experiment was performed using EASY-nLC 1000 system (Thermo Fisher Scientific) coupled to *QExactive* (Thermo Fisher Scientific) equipped with nanoelectrospray ion source. The samples were processed and analyzed with Proteome Discoverer (v2.2) against the PlasmoDB database.

### Reverse phase- high performance liquid chromatography

The homogeneity of tag-free CyRPA was determined by reverse phase high pressure liquid chromatography (RP-HPLC) using a C-18 column (Phenomenex Luna). Around 100 μg of 0.3 M tag-free CyRPA IEx elute was injected into the column. The gradient profile was developed using buffer A (0.1% triflouroacetic acid in water) and buffer B (0.1% triflouroacetic acid in acetonitrile) as follows: 0 min, 95% Buffer A, 5% Buffer B; 45 min, 5% Buffer A, 95% Buffer B.

### Ellman’s assay and endotoxin detection

Ellman’s test for free cysteines detection and endotoxin presence was done using Ellman Reagent (Sigma) and Pierce LAL Chromogenic Endotoxin Quantitation Kit (Thermo Scientific) as per manufacturer’s protocol.

### Animal immunizations

Mice and rabbit immunization protocols were approved by the Institutional Animal Ethics Committee (IAEC) and carried out in accordance with all applicable regulations at Jawaharlal Nehru University (JNU). All mice experiments used 6-8 weeks old female BALB/c mice (n=6 per group) and rabbit experiments used 3-4 months old female New Zealand White (NZW) rabbits (n=1 per group). Proteins in buffer (25 μg/dose in mice; 100 μg/dose in rabbits) were formulated with the adjuvants. AddaVax (MF-59 like squalene adjuvant) (InvivoGen), Alhydrogel (2%) (Brenntag Biosector), Montanide ISA 720 (Seppic) and complete and incomplete Freund’s adjuvants (CFA/IFA) (Sigma) was used in the mice and rabbit experiments. AddaVax was mixed with antigen 1:1 (v/v) by gentle pipetting (avoid frothing) followed by 10 min incubation before injection for a short period of time. Alhydrogel (500 μg Al^3+^/dose in mice; 850 μg Al^3+^/dose in rabbits) was combined with antigen and spun at 4°C for 30 minutes before administration to sediment protein adsorbed to Alhydrogel and analysis confirmed that >99.9% of the protein vaccine was adsorbed. CFA/IFA were mixed vigorously with antigen in 1:1 (v/v) through vortexing for 10 mins. Montanide ISA 720 was added to antigen in a 7:3 ratio and emulsified using two-syringe method for 10 minutes following manufacturer’s instructions. All immunizations were administered intramuscularly (im) in a final volume of 100 μL/dose in mice and 500 μL/dose in rabbits on days 0, 28 and 56. Serum was prepared from bleeds taken 10 days before immunization (pre-bleeds) and following 2-weeks after every immunization i.e. on day 14, 42 and 70 (or 145 in case of CyRPA/Alhydrogel, CyRPA/AddaVax and CyRPA/Montanide ISA 720 rabbit immunizations). For adjuvant controls, adjuvants were administered alone in mice at the same concentration as used in vaccine formulations as per the immunization schedule.

### Sample collection and ethics approval

Plasma samples from *P. falciparum*-infected patients were collected from Balaghat district in Madhya Pradesh by the National Institute of Research and Tribal Health (NIRTH) as described previously (23). Samples were acquired from consenting human subjects by finger-prick and *P. falciparum* infection was verified *via* detection of *P. falciparum* specific HRP-2 antigen (SD Bioline Malaria Antigen Pf/Pv; Bio Standard Diagnostics) and *via* microscopic examination of Geimsa-stained blood smears. Patients were treated for *P. falciparum* as per the drug policy of the National Vector Borne Disease Control Programme. All ethical approvals were obtained from the institutional ethical review committees, as per guidelines laid out by the Indian Council of Medical Research (ICMR), Government of India.

### Immunoblotting and ELISA

Western blotting was done as described previously (16, 23). *P. falciparum* 3D7 synchronised late-stage schizonts were harvested after which erythrocytes were lysed with 0.05% (w/v) saponin followed by lysis of parasite pellet in RIPA buffer (Sigma) with 2X protease inhibitor cocktail (Roche). Briefly, 3D7 parasite lysate, purified protein or co-immunoprecipitated elutes were transferred onto the nitrocellulose membrane overnight at 20 V or for 2 hours at 180 mA in Tris-glycine buffer at 4°C. The membrane was blocked with 5% skim milk in 1x phosphate-buffered saline (PBS), followed by incubation either with anti-CyRPA (tag-free) polyclonal sera (1:250), anti-RH5ΔNL polyclonal sera (1:250) or anti-Ripr C polyclonal sera (1:250), all raised in rabbit for 1 h. The membrane was then incubated with horseradish peroxidase (HRP)-conjugated anti-rabbit secondary IgG (1:3,000; Sigma) for 1 h. The Western blot was developed using SuperSignal™ West Pico PLUS Chemiluminescent Substrate (Thermo Scientific) or 3,39-diaminobenzidine (DAB) (1 mg/mL; Sigma) in the presence of hydrogen peroxide. After each incubation step, the membrane was washed thrice with 1x PBST (1xPBS + 0.05% Tween 20) (and also followed twice by 1xPBS after secondary antibody incubation step). All steps were performed at room temperature. For immunoblotting in the nonreduced state, the antigen was not treated with β-mercaptoethanol or heated.

The procedure used for performing ELISA was same as that previously reported (23, 34). In brief, 200 ng of antigen was coated in a 96-well microtiter plate (Nunc Maxisorp) in carbonate-bicarbonate buffer (pH 8.3) (Sigma) at 4°C overnight. The next day, plates were blocked with 2% skim milk prepared in 1xPBS at 37°C for 2 h. Plates were washed thrice with 1xPBST and primary antibody preparations of anti-CyRPA polyclonal mice/rabbit sera (different dilutions), individual human serum samples (1:200) or CyRPA monoclonal antibody (mAb) c10 (15 μg/mL) were added and incubated at 37°C for 1 h. Plates were washed thrice with 1xPBST and incubated with HRP-conjugated anti-mouse (1:10,000; Sigma), anti-rabbit (1:10,000; Sigma), or anti-human (1:10,000; Sigma) secondary antibody for 1 h at 37°C. Plates were washed thrice with 1x PBST and twice with 1xPBS, followed by development of the reaction using the substrate O-phenylenediamine dihydrochloride (OPD) (1 mg/ml; Sigma) prepared in phosphate-citrate buffer (Sigma) in the presence of hydrogen peroxide. After 30 min of incubation with the substrate, the reaction was stopped by adding 1 M sulfuric acid, followed by measurement of optical density (OD) at 490 nm in a microplate reader (Molecular Devices).

In ELISA with human sera samples, CyRPA antibody titers were measured in sera of 42 malaria infected individuals from a malaria endemic region in central India (Balaghat, MP). Samples from six malaria naïve individuals were used as negative controls. A sample was said to be a positive responder if its OD value was greater than the mean of the ODs of malaria naïve serum samples plus three times the standard deviation of their mean. Any sample with a value below this was described as a negative responder. For ELISA in the reduced state, the antigen was pre-treated with 5% β-mercaptoethanol and heated before coating, as reported previously (23).

In protein-protein ELISA assay to check interaction between RH5 and CyRPA, RH5 was coated (bait) at 200 ng along with negative control (EBA-175) and CyRPA was added (prey) at different amounts varying from 0.5-10 μg [or different concentrations (5 μg/ml to 100 μg/ml)] in an additional incubation step at 37°C for 1 h after which the plates were washed and probed with anti-CyRPA rabbit sera (1:5000) followed by washing and secondary antibody incubation as described above. Reciprocal interaction where CyRPA was coated and RH5 was added as prey was also performed (data not shown).

### Cytometric bead arrays for mouse serum cytokines

The levels of cytokines by cytometric bead array (CBA) kit, according to the manufacturer’s protocol (BD Biosciences). The data was acquired on FACS Aria Fusion (BD Biosciences) and analysed by FCAP Array software.

### *P. falciparum* blood stage culture and *in vitro* growth inhibition assay

*P. falciparum* culture and invasion inhibition assay experiments were done as described previously (23). *P. falciparum* parasite strains 3D7, 7G8, Dd2, FVO and HB3 were cultured and maintained under mixed gas environment (5% CO_2_, 5% O_2_ and 90% N_2_) at 37°C in O^+^ erythrocytes and RPMI 1640 medium supplemented with 0.5% Albumax, 0.2% sodium bicarbonate and gentamicin (10 μg/ml). *P. falciparum* cultures were synchronized by sorbitol and percoll treatments (35). For invasion inhibition assays, ring-stage malaria parasites were allowed to mature through to the schizont stage. Hematocrit and parasitemia were adjusted to 2% and 0.5%, respectively. Purified IgG from rabbit sera was added to the parasite culture in 96-well plates at various concentrations. After single cycle of invasion (40-44 h), parasites were stained with ethidium-bromide and parasitemia was measured by flow cytometry using FACS Aria Fusion (BD Biosciences). GIA was calculated with respect to erythrocyte invasion of parasite in the presence of control rabbit pre-immune IgG.

### Statistical analysis

Statistical calculations were done using GraphPad Prism Software Version 9. To assess difference between reducing and non-reducing protein ELISA OD values, unpaired t-test was performed. Mann-Whitney U test was used to calculate statistical significance in human immunogenicity analysis. Antibody titers induced by immunization, T_H_1 and T_H_2 responses and growth inhibition assay were analysed by a two-way ANOVA with Bonferroni post-hoc testing. In all cases, differences with a probability (*p)* value of <0.05 were considered significant.

## Results

### Optimization of the recombinant expression of tag-free CyRPA in *E. coli*

**Figure 1:**
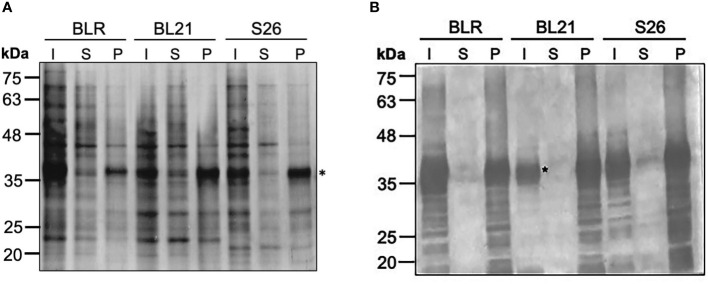
Optimization of the Expression of Tag-free CyRPA. **(A)** Different *E. coli* strains- BLR (DE3), BL21 (DE3) and *Shuffle* 26 (DE3) were tested for the expression of tag-free CyRPA. CyRPA expression was analyzed in the induced samples both in the supernatant and pellet fraction by Coomassie stained reducing SDS-PAGE. CyRPA was observed in all the three strains with BL21 (DE3) inducing the highest expression. **(B)** The expression of tag-free CyRPA as shown in **(A)** was confirmed by immunoblotting using anti-CyRPA rabbit polyclonal sera. Consistent with SDS-PAGE results, anti-CyRPA sera confirmed tag-free CyRPA expression in all the three *E. coli* strains. * indicating the position of CyRPA. I, Induced; S, Supernatant; P, Pellet; S26, Shuffle 26.

The CyRPA encoding gene sequence of the *P. falciparum* 3D7 clone was codon-optimised for expression in *E. coli*. Specific primers were used to PCR amplify and insert a stop codon to the 3’ end of the gene for sub-cloning into pET24b expression vector and avoid CyRPA expression with hexa-histidine tag. Several *E. coli* expression cell strains namely, BLR (DE3), BL21 (DE3) and Shuffle 26 (DE3) were screened at different IPTG concentrations and post-induction temperature (37°C and 16°C), to identify the optimal condition for maximal protein expression in soluble form or inclusion bodies at a 10 ml test tube scale. A high level of CyRPA expression was induced in all the three *E. coli* strains with 1 mM IPTG at 37°C, however, the protein was majorly found in inclusion bodies (IBs), as confirmed through SDS-PAGE analysis and immunoblotting using CyRPA specific serum (**Figure 1**). In an attempt to obtain soluble form of CyRPA, we used an *E. coli* co-expression system that co-expressed a heterologous chaperone (GroEL-ES) along with the protein of interest. Four *E. coli* strains, BL21 (DE3), C41 (DE3), Shuffle 26 (DE3) and Origami 2 (DE3) expressing the GroEL-ES chaperone were screened (**Supplementary Figure S1**). We observed a small level of supernatant CyRPA expression in all cells, however, our attempts to enrich the protein from this fraction were unsuccessful (data not shown).

We therefore chose to purify the protein from inclusion bodies and selected BL21 (DE3) for the scale-up of the protein production because of its fast-growing capability relative to other *E. coli* strains that would allow to minimize the time-period to reach induction time and achieve high biomass.

### Process development for the large-scale production of tag-free CyRPA

**Figure 2:**
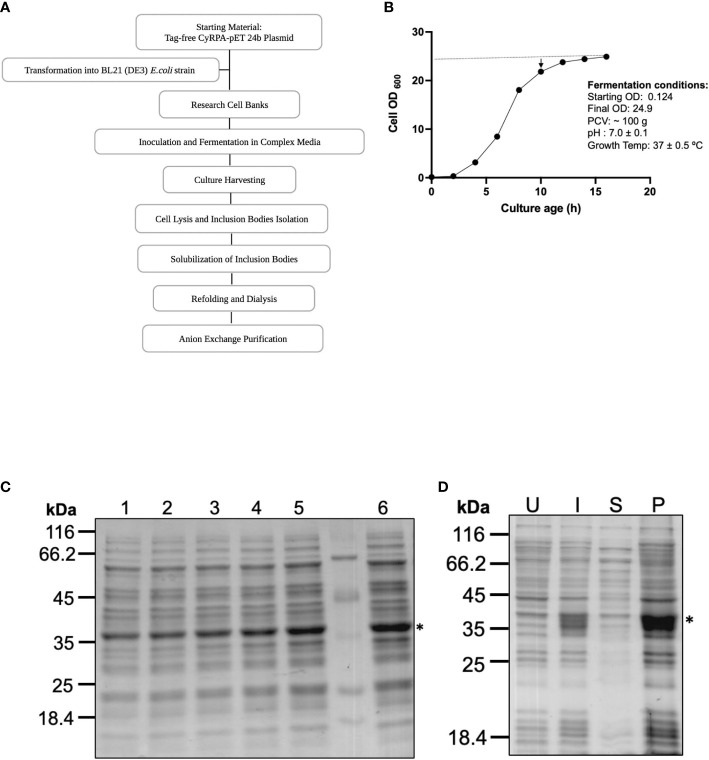
Process Development for Large Scale Production of Tag-free CyRPA. **(A)** Flow chart depicting the steps involved in the process development of tag-free CyRPA. **(B)** Time-point graph showing the entire 16 hours long fermentation run for CyRPA expressing BL21 (DE3) cells biomass production. Arrowhead indicates induction time point after 10 hours of culture growth. At this point, culture has started to enter the stationary phase. Dotted line shows that a constant pH was maintained throughout fermentation process. **(C)** CyRPA expression was monitored each hour by SDS-PAGE, which confirmed protein expression at different time points (1,2,3,4,5 and 6 hours) after induction. **(D)** Post fermentation, the expression of CyRPA was checked by reducing SDS-PAGE in uninduced, induced, supernatant and pellet fractions after cell lysis. Consistent with small scale results, expression can be found in pellet fraction or inclusion bodies. * indicating the position of CyRPA. UI, Uninduced; I, Induced; S, Supernatant; P, Pellet.

An upstream 4 L batch fermentation and downstream purification process was optimized that could be translated for carrying out human clinical studies with a CyRPA based vaccine (**Figure 2A**). For these studies, we replaced the Luria-Bertani broth growth medium with another media (here named as complex media) that was devoid of any component of animal origin to avoid contamination of the final purified product. During fermentation process, the pH of the culture was continuously monitored and maintained at ~7.0. The IPTG induction was given towards the end of the log phase, at an OD of ~21 when the growth of the cells had stabilized and began to enter the stationary phase (**Figure 2B**). Post-induction, fermentation was run for another 6 h at 37°C and CyRPA expression was monitored by SDS-PAGE (**Figures 2C, D**). After completion of the fermentation run, cells were centrifuged and a total biomass of 100 g was obtained and stored at -80°C until further use.

**Figure 3:**
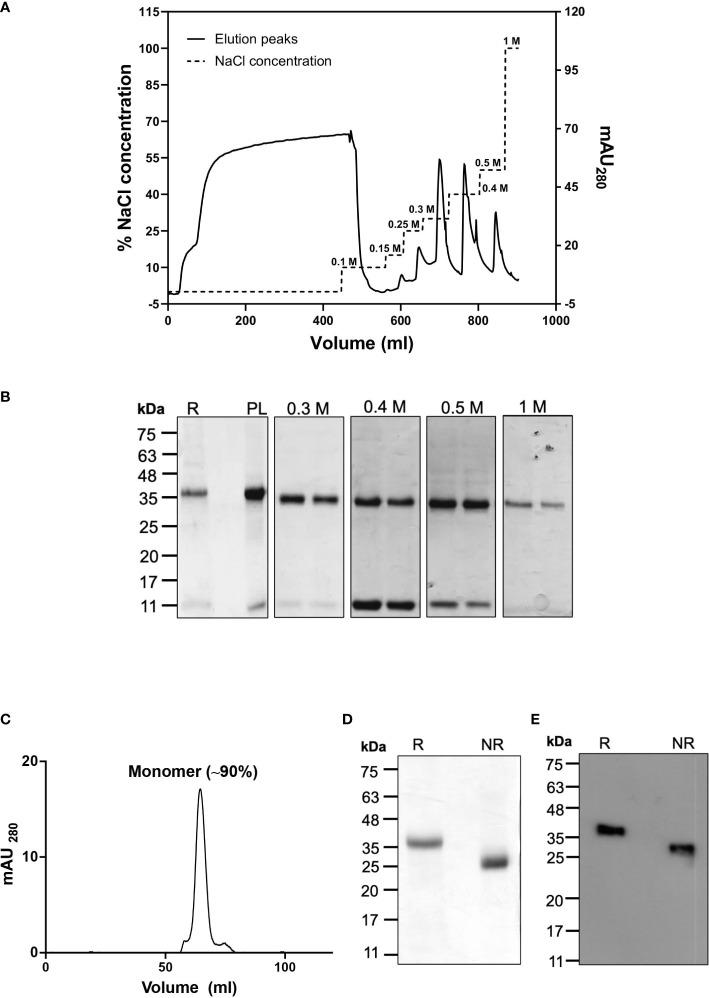
Purification of Tag-free CyRPA by Anion-Exchange Chromatography **(A)** After refolding, tag-free CyRPA was dialysed and subjected to ion exchange purification on an AKTA system using anion exchanger Q-sepharose beads. Column was equilibrated with a salt free Tris buffer, then dialyzed CyRPA was loaded onto the column, followed by washing (0.1 M NaCl) and elution (0.15, 0.25, 0.3, 0.4, 0.5 and 1 M NaCl). Solid line indicates elution peaks monitored at 280 nm and dashed lines show percent NaCl concentration used for CyRPA elution with 1 M representing the 100% concentration. **(B)** Expression of CyRPA after refolding (Refolded [R]), dialysis (dialysed or Preload [PL]) and elution was analyzed in reducing SDS-PAGE stained with Coomassie blue. The PL was loaded onto anion exchange column and after washing, elutes were collected and protein was observed at 0.3, 0.4, 0.5 and 1 M. No protein band was observed at 0.15 and 0.25 M NaCl concentration (data not shown) **(C)** Purified 0.3 M ion exchange elutes were analyzed by size exclusion chromatography that shows a major peak at ~63 ml that corresponds to the elution volume of a 40 kDa protein, thus, confirming that the 0.3 M ion-exchange fraction consisted of CyRPA monomer. The molecular weight of the eluted protein was determined using the calibration curve calculated by running protein molecular weight standards (**Supplementary Figure S3D**) **(D, E)** The monomeric CyRPA eluted at 0.3 M NaCl concentration in ion-exchange was analyzed under reducing and non-reducing conditions by Coomassie stained SDS-PAGE gel **(D)** and immunoblotting using anti-CyRPA rabbit polyclonal sera **(E)**. The resulting shift between reduced and non-reduced CyRPA in the Coomassie stained gel and immunoblot confirms that the recombinant protein was refolded successfully. This 0.3 M NaCl elute fraction was used as the final protein in subsequent experiments. mAU_280_, milli-absorbance unit; R, Reducing; NR, Non-reducing.

For the downstream processing, the biomass was sonicated to collect the inclusion bodies, which were ~50% of the total cell biomass. The inclusion bodies were then washed and cleaned to remove endotoxin, cellular debris and unwanted protein impurities by treatment with endotoxin removal buffer and a detergent-based buffer. The washed IBs were solubilised in buffer which was used for setting up the refolding by the rapid dilution method. After refolding and dialysis, the sample was immediately subjected to anion-exchange chromatography under cold conditions and highly purified CyRPA fractions were collected using a step gradient of different NaCl concentrations (**Figure 3A**). CyRPA elution started at 0.3 M NaCl concentration and continued till 1 M (**Figures 3A, B**). However, the majority of the monomeric fraction of CyRPA (~90%) got eluted at 0.3 M NaCl concentration, while at the higher elution concentrations of NaCl, a mixture of CyRPA monomer and oligomer was observed (**Supplementary Figure S2**).

The multimeric state of the eluted fractions of CyRPA was further analysed by size exclusion chromatography, which confirmed that the 0.3 M NaCl fractions of CyRPA were indeed monomeric (**Figure 3C** and **Supplementary Figure S3**). A mobility shift between reduced and non-reduced samples of the eluted CyRPA fraction was observed when run for a longer time in SDS-PAGE thus confirming the successful refolding of the protein (**Figure 3D**). The identity of the purified CyRPA protein was confirmed by mass spectrometry (**Supplementary Table S1**) and by immunoblotting using anti-CyRPA sera (**Figure 3E**). A fragment of ~12 kDa was observed in the Coomassie gel and immunoblots of CyRPA purified fractions, indicating that this could be a processed fragment of CyRPA. Using mass spectrometry, we confirmed that this fragment was indeed a processed part of CyRPA (**Supplementary Table S2**).

The yield of our fermentation process at the purified protein level was found to be 12.5 mg/L from a biomass of 25 g/L. To ascertain the consistency and reproducibility of our scale-up process, the entire protocol from upstream to downstream was repeated thrice and yielded similar results. For the subsequent studies, we used the 0.3 M NaCl anion-exchange fraction of CyRPA as it was found to be monomeric.

### Purified recombinant tag-free CyRPA mimics native CyRPA in structure and function

**Figure 4:**
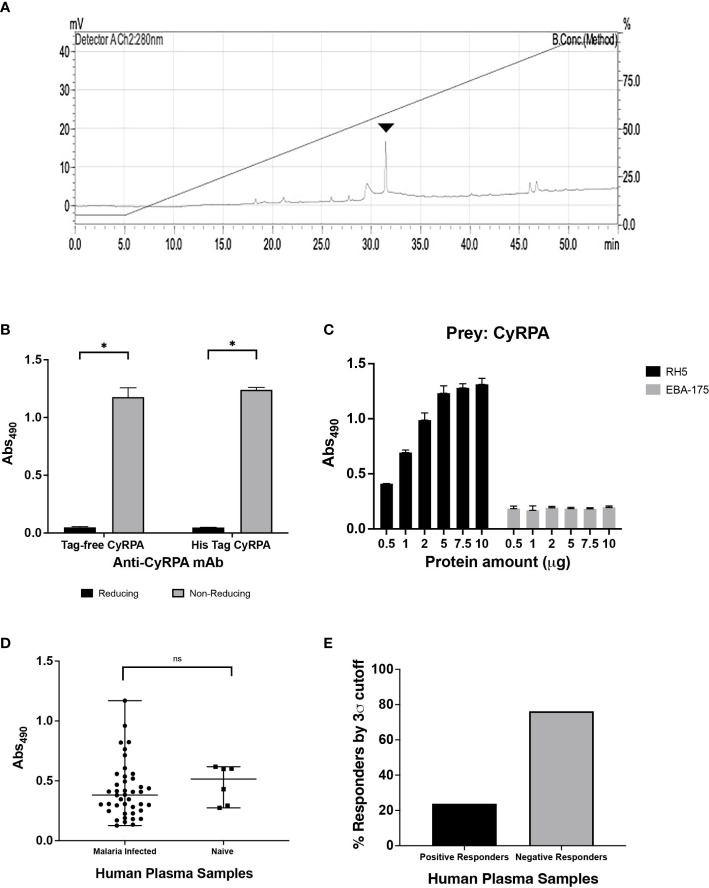
Structural and Functional Integrity of Recombinant Tag-free CyRPA. **(A)** Reverse Phase-High Performance Liquid Chromatography (RP-HPLC) analysis of the final purified recombinant tag free CyRPA showed a symmetrical peak depicting homogeneous preparation of the protein with >90% purity. Arrowhead shows the peak. **(B)** Enzyme-linked immunosorbent assay (ELISA) of tag-free CyRPA and (6-His) tagged CyRPA using conformational CyRPA monoclonal antibody (c10) under reducing and non-reducing conditions depicts recognition of non-reduced recombinant proteins by the antibody. The bars and error bars show mean and standard error values respectively. The P values were calculated by unpaired t-tests. **(C)** ELISA based interaction study between purified tag-free CyRPA and RH5 was performed. RH5 (bait) and EBA-175 RIII-V (used as negative control) were coated and CyRPA (prey) was added at different concentrations (5 μg/ml to 100 μg/ml). The interaction intensity between CyRPA and RH5 increased with increase in CyRPA concentration, while no interaction was observed between CyRPA and EBA-175 RIII-V. The bars and error bars show mean and standard error values for each protein amount respectively. **(D, E)** Seropositivity of CyRPA was analyzed in plasma from 42 malaria-infected individuals obtained from a malaria endemic region, Balaghat (Madhya Pradesh, India) by ELISA. CyRPA exhibited detectable levels of antibodies with absolute ELISA values ranging from ~ 0.1 to 1.1 optical density (OD) units. Sera isolated from six malaria naïve individuals were used as controls and to calculate the cut-off value, which is the mean OD of control malaria-naive samples plus three times standard deviation. Graph shows median values and ranges. The P values were calculated by Mann-Whitney U test. **(E)** CyRPA was poorly immunogenic with <30% of the samples showing a positive response to the antigen. A sample with ELISA OD value above the cut-off was considered a positive responder, while samples with OD values below the cut-off were defined as negative responders. mV, millivolt; Abs_490_, absorbance at 490 nm. *P≤0.05; ns- non-significant.

To evaluate the quality and structural integrity of the purified tag-free CyRPA, we first analysed it using Reversed-Phase HPLC technique that showed a single major peak corresponding to CyRPA thus indicating towards the highly homogenous nature of our purified sample (>90% purity) (**Figure 4A**). LAL test analysis for the levels of endotoxin in our recombinant CyRPA preparation showed to be ~1.3 EU/mg, which is within the acceptable limits for human use of a vaccine (36).

CyRPA contains 10 cysteine residues that are involved in intra-disulfides formation necessary for maintaining the protein’s structure. Ellman test analysis of our CyRPA preparation showed that it has no free sulfhydryl group thus confirming that all cysteines are involved in disulfide bond formation as it exists in the native protein. To further confirm if the purified CyRPA has correctly folded, we analysed the ability of a conformation-specific CyRPA monoclonal antibody (c10) to recognize the purified protein using Enzyme-Linked Immunosorbent Assay (ELISA). The monoclonal antibody readily detected the recombinant CyRPA under non-reducing conditions but failed to detect reduced CyRPA (**Figure 4B**). The purified CyRPA protein also interacted directly with its interacting partner, RH5, in an ELISA based protein-protein interaction assay, further confirming the correct conformation of the protein (**Figure 4C**).

Seropositivity analysis of our recombinant CyRPA preparation showed that it was recognizable by sera collected from malaria-infected individuals residing in the malaria endemic region, Balaghat in Madhya Pradesh, India (**Figure 4D**). CyRPA exhibited low immunogenicity (ELISA OD units ranging from ~0.1 to 1.1) with only ~30% sera samples being positive for the antigen (**Figure 4E**), which is consistent with previous reports from our lab and others (18, 21, 23, 24). Together, these results show that the purified recombinant tag-free CyRPA has similar structural and functional properties to that of the native antigen.

### Tag-free CyRPA elicits potent parasite neutralizing antibodies

**Figure 5:**
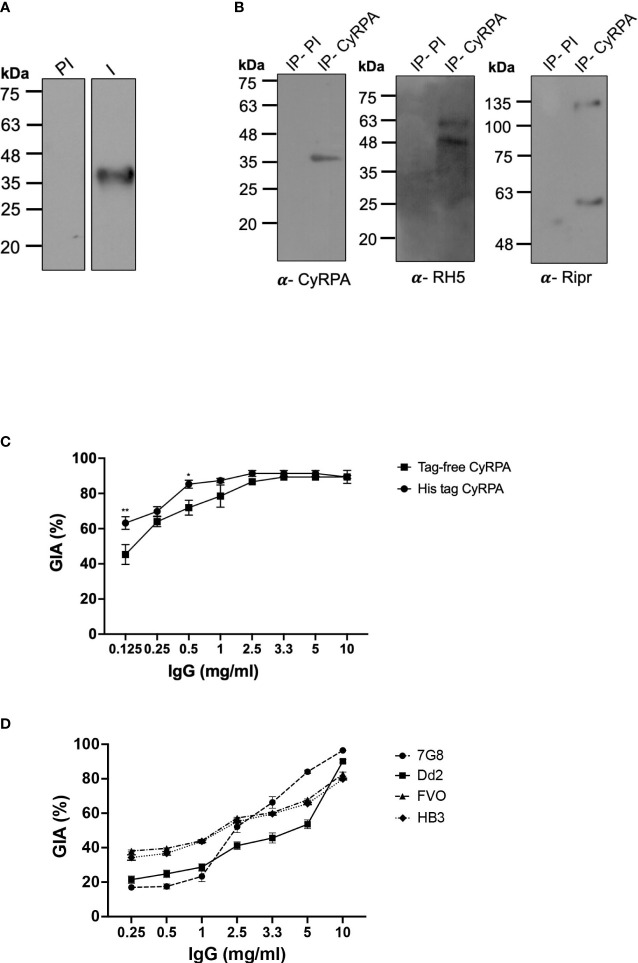
Parasite Neutralizing Ability of Antibodies Targeting Tag- Free CyRPA. **(A)** Antibodies raised against tag-free CyRPA were evaluated for specificity by their ability to recognize native CyRPA protein. Schizont extracts from 3D7 parasites were probed with anti-CyRPA immune (I) and pre-immune (PI) rabbit polyclonal sera through immunoblotting. Immune sera specifically detected a band at ~40 kDa corresponding to CyRPA, while no signal was detected with the pre-immune sera. **(B)** To confirm the ability of CyRPA rabbit antibodies to detect the native protein in its native form, co-immunoprecipitation experiment was performed with 3D7 schizont lysate. As shown, the co-immunoprecipitated components of the multi-protein complex (RH5, Ripr and CyRPA) were successfully detected using their respective specific rabbit antibodies in immunoblotting. Pre-immune sera, used as a negative control, could not coimmunoprecipitate the multiprotein complex. **(C)** Purified rabbit total IgG against tag-free CyRPA were tested in the standard *in vitro* Growth Inhibitory Assay (GIA) against 3D7 clone in a one-cycle assay. The antibodies exhibited highly potent dose-dependent parasite neutralization reaching saturation at 2.5 mg/ml with ~90% inhibition levels. The antibody efficacy was similar to those against (6-His) tagged CyRPA that was used as a positive control except at low concentrations of 0.125 and 0.5 mg/ml. Data represents the average of 2 independent experiments conducted in duplicate. The error bars represent the standard error between the 2 independent GIA experiments. The P values were calculated by two-way ANOVA with Bonferroni post-hoc testing. **(D)** Total IgG against tag-free CyRPA was tested against four *P. falciparum* clones- 7G8, Dd2, FVO and HB3, in a one-cycle GIA. A robust strain transcending parasite neutralization activity was observed by the antibodies across the four strains with inhibition levels reaching to ~80% to 96%. Data represents the average of 2 independent experiments conducted in duplicate. The error bars represent the standard error between the 2 independent GIA experiments. I, Immune; PI, Pre-immune; IP, Immunoprecipitate.

To evaluate the ability of recombinant tag-free CyRPA to elicit functional antibodies, mice and rabbits were immunized with the antigen formulated in Freund’s complete and incomplete adjuvant (CFA/IFA) that induced a highly immunogenic antibody response (endpoint titers:- mice- ~25,60,000; rabbits- ~51,20,000). The antibodies specifically recognised native CyRPA in schizont lysate of 3D7 parasites (**Figure 5A**). To further confirm the ability of these antibodies to recognise CyRPA in its native state, we performed co-immunoprecipitation studies using parasite schizont lysate. The antibodies successfully coimmunoprecipitated the RH5/Ripr/CyRPA complex, as confirmed by immunoblotting of the coimmunoprecipitated sample using respective antigen specific antibodies (**Figure 5B**).

We have earlier shown that antibodies raised against (6-His) tagged CyRPA exhibit highly potent cross-strain parasite neutralization (16). To ensure that removal of hexahistidine tag did not compromise the ability of tag-free CyRPA in inducing similarly potent neutralizing antibodies, we carried out a head-to-head comparison of the efficacy of antibodies targeting the (6-His) tagged and tag-free CyRPA. Both antibodies showed potent parasite neutralization reaching saturation at just 2.5 mg/ml total IgG concentration with inhibition levels of ~90% (**Figure 5C**) with similar end-point titers (~51,20,000) (**Supplementary Figure S4**). However on comparing the inhibition at each IgG concentration, only at 0.125 and 0.5 mg/ml, (6-His) tagged CyRPA antibodies exhibited significantly higher inhibition than tag-free CyRPA (^**^P ≤ 0.01 and ^*^P ≤ 0.05 respectively).

We next evaluated the strain-transcending parasite neutralizing ability of antibodies targeting the tag-free CyRPA. Four parasite strains of different geographical origins and invasion pathways viz. Dd2, HB3, 7G8 and FVO were studied. Similar to 3D7, a robust dose-dependent invasion inhibition was observed that ranged from ~80% to 96% at 10 mg/ml IgG concentration across the four strains (**Figure 5D**). These results demonstrate that tag-free CyRPA produced in a complex media at a fermenter scale induces functional antibodies that exhibit efficacious strain-transcending parasite neutralization.

Several studies have observed that oligomers can induce a better immunogenic response than the monomeric form of the antigen (37–39). Since we observed CyRPA oligomers during ion-exchange purification, we got interested to test and compare their immunogenicity and ability to induce parasite neutralizing response relative to CyRPA monomers. To this end, CyRPA oligomers-Freund’s formulation was used to immunize rabbit. Total IgG was purified from Day 70 sera (end-point titers ~25,60,000) to conduct GIA vis-à-vis with anti-CyRPA monomer antibodies (**Supplementary Figure S5A**). We observed that antibodies against the oligomeric form of CyRPA could only achieve <60% invasion inhibition at 10 mg/ml IgG concentration compared to antibodies against the CyRPA monomers (**Supplementary Figure S5B**). This data clearly showed that CyRPA monomers elicit more efficacious immune response than their oligomers. Together, these findings show that tag-free CyRPA is highly immunogenic and elicits potent cross-strain parasite neutralizing antibodies as previously shown for (6-His) tagged CyRPA.

### Tag-free CyRPA formulated with human-compatible adjuvant elicits a highly immunogenic humoral and cell-mediated response

We were interested to identify a CyRPA based vaccine formulation that could be translated for human clinical studies. Mice and rabbits were immunised with CyRPA vaccine formulated with commonly used adjuvants- Alhydrogel (or alum), Montanide ISA 720 and AddaVax to investigate their ability to induce anti-parasitic immune response.

**Figure 6:**
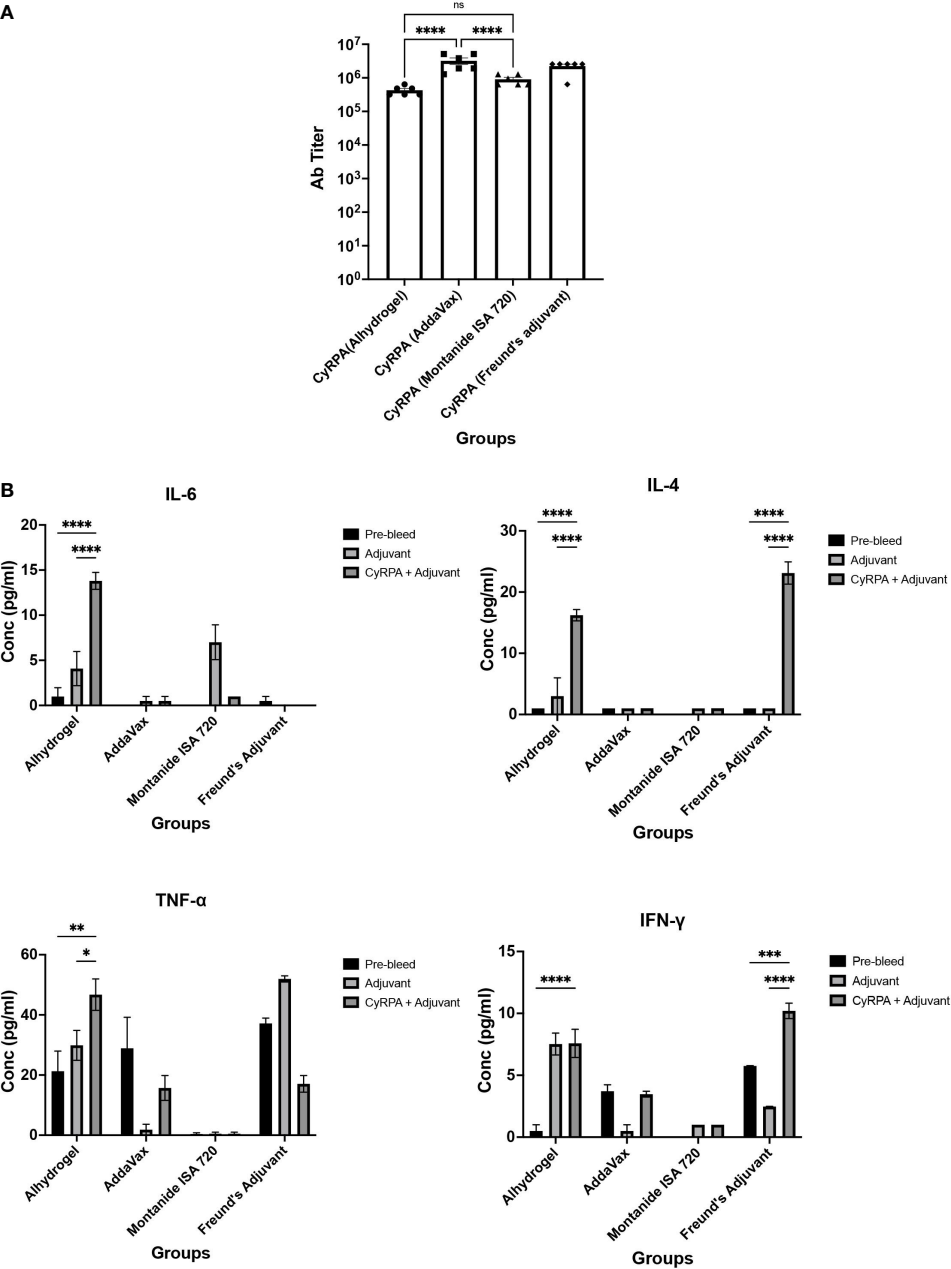
Humoral and Cellular Immune Responses against Tag-free CyRPA Vaccine Formulated with Commonly used Adjuvants. **(A)** BALB/c mice (n = 6 per group) were immunized intramuscularly with tag-free CyRPA formulated with three adjuvants- Alhydrogel, AddaVax and Montanide ISA 720 and Freund’s adjuvant (CFA/IFA) as a control. Enzyme-Linked Immunosorbent Assay (ELISA) was performed to calculate antibody titers in Day 70 sera. CyRPA (AddaVax) and CyRPA (Freund’s Adjuvant) induced high antibody titers (~32,00,000 and ~25,60,000 respectively) followed by CyRPA (Montanide ISA 720) (~6,40,000) and CyRPA (Alhydrogel) (~4,20,000). ELISA cut-offs were calculated as the mean OD_490_ of pre-bleed sera plus three times standard deviation. The bars and error bars show mean and standard error values of each vaccine group respectively. Individual mice titers are also shown. The P values were calculated by two-way ANOVA with Bonferroni post-hoc testing. **(B)** Levels of T_H_1 and T_H_2 responses were measured in BALB/c mice serum immunized with CyRPA+adjuvant or adjuvant alone by cytometric bead arrays (CBA) for mouse serum cytokines using flow-cytometry. CyRPA/Alhydrogel vaccine induced IL-4, IL-6, TNF-α when compared to the Alhydrogel only group while no detectable responses were observed for CyRPA/AddaVax and CyRPA/Montanide ISA 720 groups. However the control group- CyRPA/Freund’s Adjuvant observed increased levels of IFN-γ and IL-4. The pre-bleed, adjuvant and CyRPA+adjuvant plots refer to the cytokines detected in sera from groups of 6 mice before immunization, post-immunization of adjuvant and post-immunization of CyRPA formulation respectively. The bars and error bars show mean and standard error values of each group respectively. The P values were calculated by two-way ANOVA with Bonferroni post-hoc testing. * P≤0.05 ** P≤0.01, *** P≤0.001, **** P≤0.0001.

For immunogenicity studies, CyRPA formulations with adjuvants were immunized in groups of six female BALB/c mice with three doses (25 μg) at four-weeks interval. As a control, mice were given CyRPA/Freund’s formulation. Day 70 sera was used to quantify the vaccine-specific antibodies by ELISA. All the vaccine formulations induced a strong antibody response. CyRPA/AddaVax was most immunogenic with end-point titers in the range ~32,00,000 that were significantly higher than antibody titers induced by CyRPA/Alhydrogel (~4,20,000) and CyRPA/Montanide ISA 720 (~6,40,000) but comparable to those against CyRPA/Freund’s formulation (~25,60,000) (**Figure 6A**).

To understand the cell-mediated immune response to CyRPA vaccine adjuvanted with commonly used adjuvants, we carried out the flow cytometry-based analysis of T_H_1 and T_H_2 cytokines using the Day 70 sera from the vaccinated mice (**Figure 6B**). Sera of mice injected with adjuvant alone and un-immunized mice were used as controls in the assay. The CyRPA/Alhydrogel vaccine induced secretion of IL-4 and IL-6. While Alhydrogel is known to elicit a T_H_2 directed immune response, we also observed increased levels of TNF-α, which is a T_H_1 specific cytokine in mice immunized with CyRPA/Alhydrogel. On the other hand, we did not observe detectable levels of any cytokine in the CyRPA/AddaVax and CyRPA/Montanide ISA 720 vaccinated mice groups, except the induction of small levels of IL-6 in the Montanide ISA 720 alone group. In the CyRPA/Freund’s immunized mice, increased levels of both T_H_1 (IFN-γ) and T_H_2 (IL-4) cytokines were observed. Together, our data shows that among the three formulations of CyRPA, only CyRPA/Alhydrogel induced detectable parasite specific cytokine response.

### CyRPA/Alhydrogel antibodies exhibit most efficacious strain-transcending parasite neutralization

**Figure 7:**
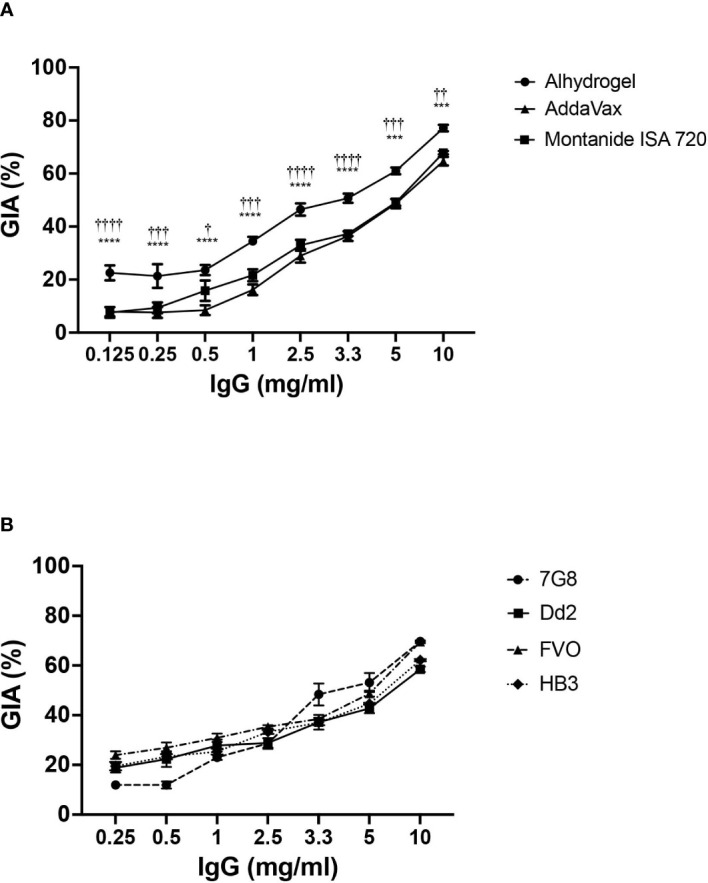
Parasite Neutralizing Efficacy of Antibodies against CyRPA Vaccine Formulated with Commonly used Adjuvants **(A)** Purified rabbit total IgG against the different tag-free CyRPA vaccine formulations (Alhydrogel, AddaVax and Montanide ISA 720) were tested in the standard *in vitro* Growth Inhibitory Assay (GIA) against 3D7 clone in a one-cycle assay. All the formulations elicited parasite neutralizing antibodies, however, anti-CyRPA/Alhydrogel IgG antibodies were the most efficacious exhibiting significantly higher inhibition levels that reached to ~80% at 10 mg/ml. Data represents the average of 2 independent experiments conducted in duplicate. The error bars represent the standard error between the 2 independent GIA experiments. The P values were calculated by two-way ANOVA with Bonferroni post-hoc testing. ^*^indicates P value difference between Alhydrogel and AddaVax groups. ^†^indicates P value difference between Alhydrogel and Montanide ISA 720 groups. **(B)** Total IgG against tag-free CyRPA/Alhydrogel was tested against four *P. falciparum* clones, 7G8, Dd2, FVO and HB3, in a one-cycle GIA. The antibodies exhibited a strain-transcending parasite neutralization across the four strains achieving the inhibition level of ~70% (7G8 and HB3) and ~60% (Dd2 and FVO). Data represents the average of 2 independent experiments conducted in duplicate. The error bars represent the standard error between the 2 independent GIA experiments. The P values were calculated by two-way ANOVA with Bonferroni post-hoc testing. *** P≤0.001, **** P≤0.0001, †† P≤0.01 ^†††^ P≤0.001, ^††††^ P≤0.0001.

To estimate the parasite neutralizing efficacy of CyRPA vaccine formulations, rabbits were immunized with the three formulations as per the protocol used for mice immunizations. Sera collected (on Day 145 instead of Day 70) after the second boost was used to purify the total IgG and perform GIA. A dose-dependent parasite neutralization was observed with antibodies against all the three formulations when tested against the 3D7 *P. falciparum* strain. Among the three vaccine formulations, CyRPA/Alhydrogel specific antibodies were most potent, exhibiting an inhibition of erythrocyte invasion of ~80% at 10 mg/ml total IgG concentration (**Figure 7A**). At the same IgG concentration, the CyRPA/AddaVax and CyRPA/Montanide ISA 720 specific antibodies achieved the highest inhibition of ~60% (^***^P ≤0.001 and ^††^ P ≤ 0.01 respectively). Since antibodies induced by CyRPA/Alhydrogel were most potent, we further evaluated their strain transcending parasite neutralizing efficacy against four other parasite strains, Dd2, HB3, 7G8 and FVO in the GIA. Like 3D7, a dose-dependent inhibition of erythrocyte invasion was observed across the four strains (**Figure 7B**). The inhibition levels reached to ~70% against 7G8 and HB3 at 10 mg/ml, while a highest inhibition of ~60% was observed against Dd2 and FVO at the same total IgG concentration. Overall, our data shows that tag-free CyRPA/Alhydrogel vaccine is a target of strain-transcending parasite neutralizing antibodies.

## Discussion

*P. falciparum* erythrocyte invasion is a highly attractive step for the identification of potent vaccine targets (12, 40). Numerous merozoite antigens have been identified to be the target of protective naturally acquired antibodies against malaria, thus suggesting towards their potential as vaccine candidates (8, 41). However, apart from RH5, no other antigen has shown promising results in clinical trial studies (42–44). Our group identified CyRPA to form an essential multiprotein complex with RH5 and Ripr during erythrocyte invasion (16). We showed CyRPA to be the target of potent *in vitro* strain-transcending parasite neutralizing antibodies that was substantiated by other groups, thus establishing CyRPA as a promising blood-stage vaccine target (17, 18, 25). Recent findings from our lab that demonstrated synergistic invasion inhibition of CyRPA based antibody combination along with the parasite neutralizing ability of naturally acquired human CyRPA antibodies further strengthened the idea of developing a CyRPA based malaria vaccine (16, 20, 23). In the present study, we have for the first time provided a workflow of a process for the successful large-scale production of CyRPA in *E. coli* and identified an efficacious human adjuvant based CyRPA vaccine formulation.

Recombinant production of CyRPA has been reported using various expression platforms including mammalian (HEK293) (18, 45–48), bacteria (*E. coli) (* 16, 18) and insect expression system (49, 50). We chose bacterial system for CyRPA expression since it has several advantages over the others: 1) ease to operate, 2) cost-effective as it allows high expression yields and easy scale-up of the process, 3) time effective due to fast growth kinetics of bacteria (51). Subunit vaccine production often involves addition of a purification tag to the target antigen to facilitate its purification. Hexa-histidine is a commonly used tag that has also been used in several malaria vaccine candidate studies (27, 52, 53). However, reports have shown presence of hexa-histidine specific antibodies as a result of immunizing hexa-histidine tagged antigen (54), hence raises safety concerns. Therefore, we adopted the strategy of producing CyRPA without any tag to meet the safety requirements of vaccine-regulatory bodies globally that recommend against the use of any extraneous amino acid sequence to a vaccine candidate.

One of the major challenges in vaccine manufacturing process is to produce a high-quality antigen with minimal cost (51, 55). Here, we have provided a single-step purification method of tag-free CyRPA with a purity of >90%. Our biochemical and biophysical assays confirmed that the purified tag-free CyRPA mimics the conformation of the native protein. Importantly, seropositivity analysis showed that the protein is recognized by sera from malaria infected patients, which further substantiated the structural and functional integrity of the purified tag-free CyRPA. In our comparative analysis of the efficacy of antibodies against CyRPA monomers and oligomers, we observed that antibodies against the CyRPA monomers induced much stronger anti-parasitic response. A possible reason to this observation could be the masking of neutralising epitopes due to oligomerization. It is noteworthy that while CyRPA oligomerization may have resulted in the reduced immunogenicity against CyRPA, co-immunization of CyRPA with other parasite antigens (RH5 and MSP-1_19_) as shown recently by our group, did not show any immune interference against any of the antigens (20). Our data combined with previous findings support the inclusion of CyRPA in a multi-component malaria vaccine but underscores the importance of producing the antigen in a monomeric form to induce most efficacious immune response.

We have previously shown that (6-His) tagged CyRPA elicits a potent strain-transcending parasite neutralizing antibody response (16). While we observed strong parasite inhibition by antibodies elicited against tag-free CyRPA that was similar to (6-His) tagged CyRPA antibodies, the latter was found to exhibit significantly higher inhibition at lower IgG concentrations. Despite these differences, both the antibodies achieved saturation at 2.5 mg/ml. This suggests that there could be slight difference in the titers of neutralizing antibodies against both the constructs that was not apparent in our comparison of their total IgG titers (**Supplementary Figure S4**). To this end, a better understanding of the effect of removal of the tag on induction of neutralising antibodies could be achieved by direct comparison of affinity purified CyRPA specific antibodies instead of CyRPA specific total IgG. Besides, future studies to understand the quality of two types of antibodies should also consider factors such as avidity, affinity, IgG subtype to understand possible mechanisms that can lead to the observed differences in parasite neutralization at lower IgG concentrations between the two antibodies.

One of the primary objectives of this study was to identify the human-compatible adjuvant based CyRPA vaccine formulation that induces robust parasite neutralizing antibody response. Our screening process included three adjuvants, Alhydrogel, AddaVax and Montanide ISA 720 that have also been tested with different experimental vaccines (56–59). All the three adjuvant formulations thus tested induced a robust immunogenic response with CyRPA/AddaVax eliciting the highest titers. However, the high titers induced by the CyRPA/AddaVax did not get translated into an equally potent anti-parasite response as antibodies induced by the formulation exhibited only moderate invasion-inhibitory activity. Interestingly, the CyRPA/Alhydrogel formulation that had the lowest immunogenicity among the three formulations, induced the most potent parasite neutralizing antibody response. The ability of CyRPA/Alhydrogel formulation to elicit a stronger neutralizing response despite having the lowest immunogenicity could be attributed to its ability to also induce a T_H_1 and T_H_2 mediated response which was not seen with either Montanide ISA 720 or AddaVax formulations. One immediate effect of T_H_1 and T_H_2 response is the IgG subclass switching that may be responsible for higher neutralizing efficacy of antibodies induced by the Alhydrogel formulation compared to Montanide ISA 720 or AddaVax formulations. This data clearly indicate that a high antibody titer does not necessarily suggest an equally strong neutralizing antibody response. The parasite neutralizing activity of antibodies induced by CyRPA/Alhydrogel was relatively lower than those induced by CyRPA/Freund’s formulation. However, one major reason for this variation could be due to different time-points for collection of rabbit sera that was used to purify total IgG for GIA. For the CyRPA/Freund’s formulation, the serum was collected at Day 70 i.e., after 2 weeks of 2^nd^ boost, while, due to COVID-19 lockdown resulting in the shutting down of the campus, the corresponding serum for the CyRPA/Alhydrogel formulation was collected on Day 145^th^, which effectively is ~3 months post 2^nd^ boost. It is likely that this long gap might have caused waning of antibody titers thus affecting the immunogenicity as well as the neutralization efficacy of the antibodies. It is noteworthy that inhibition levels of 60-70% were detected across multiple parasite strains with the Day 145 antibodies induced by the CyRPA/Alhydrogel formulation. The presence of broadly neutralizing antibodies in high titers till Day 145 could be attributed to the ability of Alhydrogel to enhance humoral immunity by formation of a “depot” by which antigen is slowly released to increase antibody production called the “repository effect” (60). Other well-known mechanisms for immuno-stimulation by Alhydrogel are pro-phagocytic effect and activation of the pro-inflammatory NLRP3 pathway (60–62). This indicates that Alhydrogel increases persistence of CyRPA thus highlighting the potential of CyRPA/Alhydrogel formulation in inducing a long-lasting immune response.

Alhydrogel (aluminium hydroxide) is the most commonly used human adjuvant with a well-documented profile of inducing T_H_2 type responses (63, 64). Our analysis showed a mixed T_H_1 and T_H_2 response dominated by the latter against CyRPA/Alhydrogel. The unexpected T_H_1-induced cytokines can be stipulated due to intramuscular mode of immunization. Recent studies show that depending on the vaccination route especially when injected intramuscularly in mice, Aluminium hydroxide-based adjuvants can enhance both T_H_1 as well as T_H_2 cellular responses which might also explain the results observed in our study (60). Besides, in malaria, a T_H_1 type response is associated with protection such that IFN-γ and TNF-α have been observed to provide resistance against Plasmodium infection (65, 66). Therefore, it is pertinent to speculate that the mixed T_H_1 and T_H_2 response against CyRPA/Alhydrogel formulation not only could improve the quality and memory of antibody response *via* T_H_2 directed response but also facilitate in providing resistance against malaria infection through T_H_1 response.

In addition to merozoite surface antigens, several studies have also focused on parasite proteases as potential vaccine targets. One such vaccine is BK-SE36 that is based on the N-terminal region of SERA5. While clinical studies have shown BK-SE36 to be safe, immunogenic and provide partial protection against high parasitaemia, it was observed that the vaccine is effective only in young children and repeated vaccination led to immune tolerance in adults and older children (67–69). Several challenges therefore need to be overcome to develop a SERA5 based vaccine. While the N-terminal region of SERA5 has been found to consist of the immunodominant epitopes, the role of its other regions needs to be established, and a vaccine based on them must be tested. This becomes more important given the studies that suggest disordered regions (like the SERA5 N-terminal region) to be more enriched in polymorphic hot-spots than ordered regions (70). Besides, the other major challenge with a SERA5 vaccine is that the antigen although shown to play an important role in parasite egress is non-essential (71). Therefore, given the overlapping function of SERA protein family members, a SERA5 vaccine may have to overcome the possible functional redundancy, which is observed with various merozoite surface ligands. Given these challenges that are also associated with other promising vaccine candidates (AMA-1, MSP-1), development of a vaccine based on CyRPA, which is essential for the survival of the parasite and less prone to acquire sequence polymorphism, appears to be a positive step forward in our efforts of producing a highly effective malaria vaccine. Our study holds particular importance with respect to the implementation of a blood-stage vaccine in African regions that have limited public health resources and a poor economy. Firstly, an earlier report from the lab has shown that anti-CyRPA antibodies potently inhibited erythrocyte invasion by *P. falciparum* clinical isolates obtained from malaria endemic regions of India and Africa, which exhibited highly redundant invasion pathways (20). Secondly, analysis of CyRPA genetic diversity in worldwide clinical isolates including Africa showed presence of a single nucleotide polymorphism (SNP), thus suggesting a highly conserved nature of the antigen, unlike AMA-1 or MSP-1, which have been said to be highly polymorphic (72–74) as well as RTS,S that consists of a polymorphic C-terminal region of CSP (75). The immunogenicity profile of CyRPA during natural infection in Africa as well as India showed that it elicits poor immunogenic response (20), which suggests that the antigen is less prone to acquire SNPs and undergo immune escape. Lastly, in an immuno-epidemiological analysis of a cohort of children in Mozambique, Africa, it was found that CyRPA specific antibodies were associated with protection from malaria reinfection (24). Besides, the ability to produce CyRPA based protein-in-adjuvant vaccine in a cheap and economical process as reported here, is highly relevant for its successful implementation in Africa. Together, these findings provide strong evidence that a CyRPA based vaccine could generate an efficacious response in the most affected malaria endemic region, Africa.

In summary, the present study reports an economical and efficient process for the production for tag-free version of the leading blood stage vaccine candidate, *Plasmodium falciparum* antigen- CyRPA in *E. coli*. Our study further demonstrates the potential of tag-free CyRPA adjuvanted with the licensed human-compatible Alhydrogel in generating efficacious parasite specific humoral and cellular response in small animals. These findings thus provide support for advancement of CyRPA based vaccines in clinical testing. Finally, our study further provides a rational basis for the evaluation of CyRPA based multiantigen combinations targeting different erythrocyte invasion ligands (RH5, Ripr) and steps (MSP-1_19_) as well as multiple parasite stages (CSP, Pfs230) with novel human adjuvants in order to meet the challenges in malaria vaccine development efforts.

## Data availability statement

The raw data supporting the conclusions of this article will be made available by the authors, without undue reservation.

## Ethics statement

The studies involving human participants were reviewed and approved by National Institute of Research on Tribal Health (NIRTH), ICMR. The patients/participants provided their written informed consent to participate in this study. The animal study was reviewed and approved by Institutional Animal Ethics Committee (IAEC), Central Laboratory Animal Resources, Jawaharlal Nehru University. Written informed consent was obtained from the individual(s) for the publication of any potentially identifiable images or data included in this article.

## Author contributions

DG and VC designed the study. AS, SM, KC and SU performed experiments. PB provided reagents. AS, SM and RT analysed the data. AS and SM wrote the manuscript. DG and VC supervised the work. All authors reviewed the manuscript. All authors contributed to the article and approved the submitted version.

## Funding

This work was supported by the following grant: Vaccine Grand Challenge Program, Department of Biotechnology (DG; VC) grant number: BT/PR5158/MED/15/79/2012. AS is the recipient of the Senior Research Fellowship of the Indian Council of Medical Research (ICMR), Government of India. SM and SU are the recipients of the Senior Research Fellowship of the Department of Biotechnology (DBT), Government of India. KC is the recipient of Senior Research Fellowship of the University Grants Commission (UGC), Government of India.

## Acknowledgments

We thank the following: Dr. Louis Miller (NIH) for providing the *P. falciparum* laboratory adapted clones; The JNU Central Laboratory Animal Resources for their technical assistance; Dr. Gagandeep Jhingan, V Proteomics for Mass Spectrometry analysis; Dr. Nitin Yadav, ICGEB and Dr. Ashwani Gautam, IIT Delhi for technical help and inputs.

## Conflict of interest

The authors declare that the research was conducted in the absence of any commercial or financial relationships that could be construed as a potential conflict of interest.

## Publisher’s note

All claims expressed in this article are solely those of the authors and do not necessarily represent those of their affiliated organizations, or those of the publisher, the editors and the reviewers. Any product that may be evaluated in this article, or claim that may be made by its manufacturer, is not guaranteed or endorsed by the publisher.
