# Supplementary figures and images for: Process development and preclinical evaluation of a major Plasmodium falciparum blood stage vaccine candidate, Cysteine-Rich Protective Antigen (CyRPA)
Source: Front Immunol. 2022 Sep 22;13:1005332. doi: 10.3389/fimmu.2022.1005332 (PMC9535676; doi:10.3389/fimmu.2022.1005332)

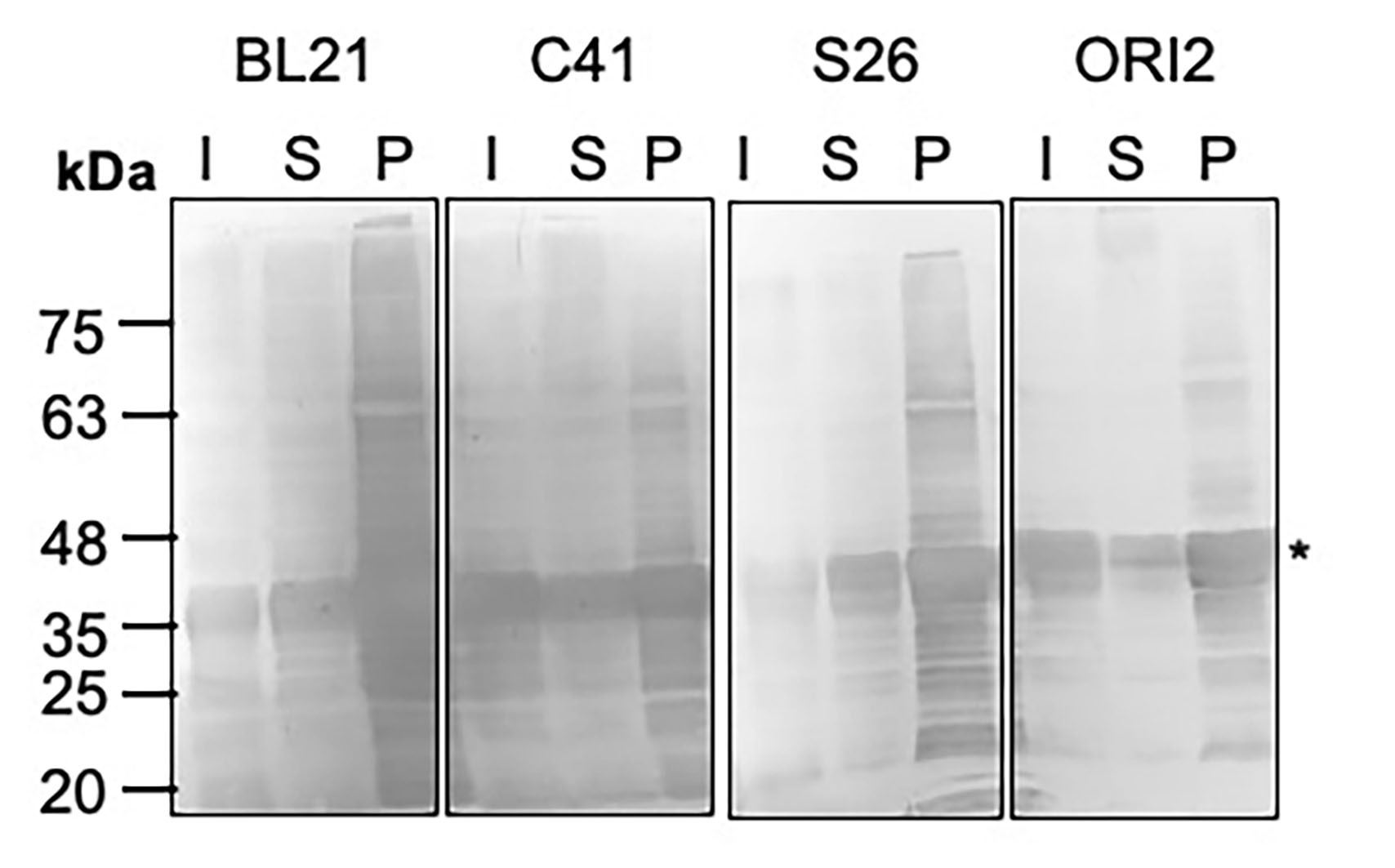

Supplement: Supplementary file 1 [file Image_1.jpeg]

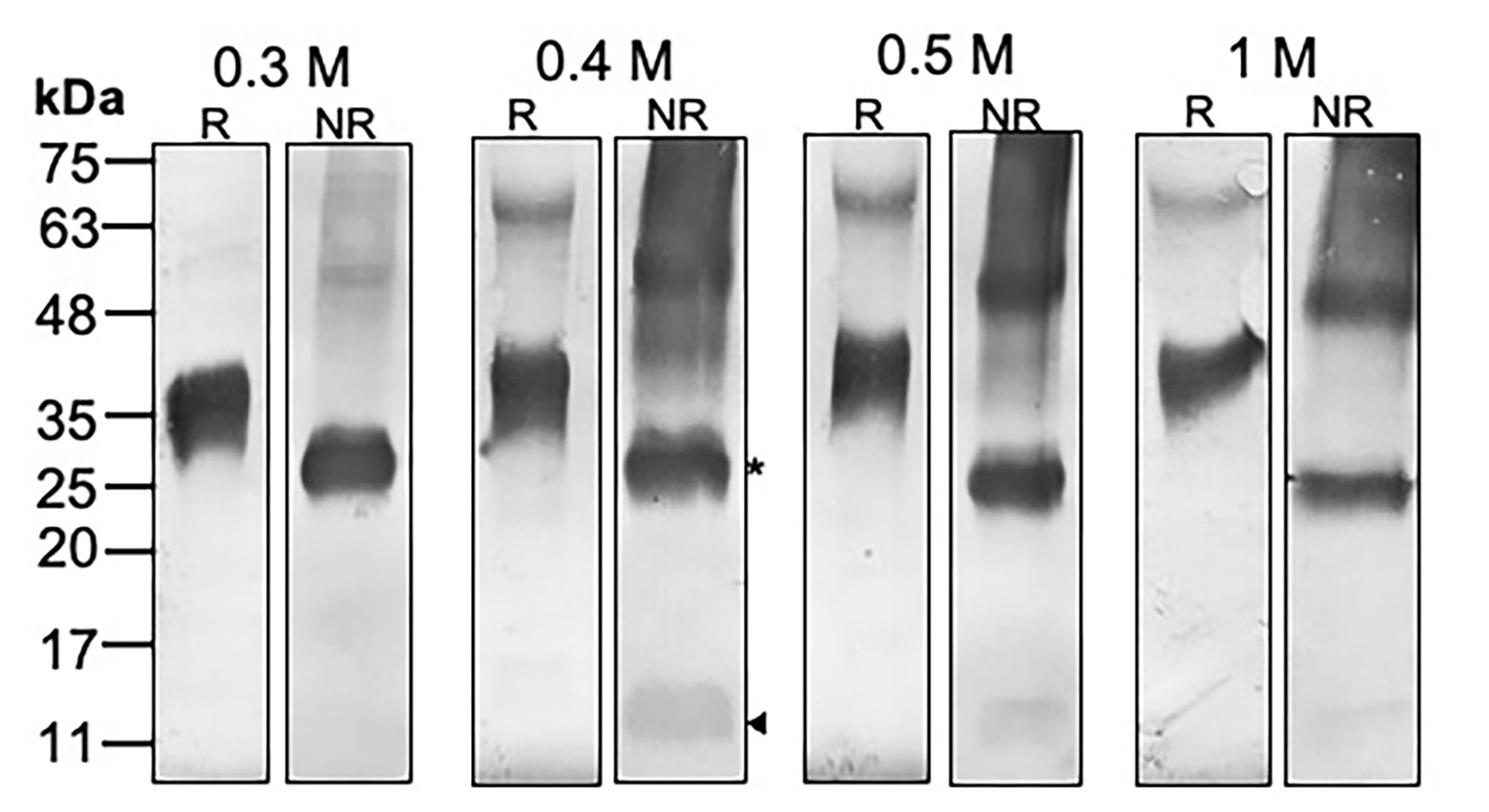

Supplement: Supplementary file 2 [file Image_2.jpeg]

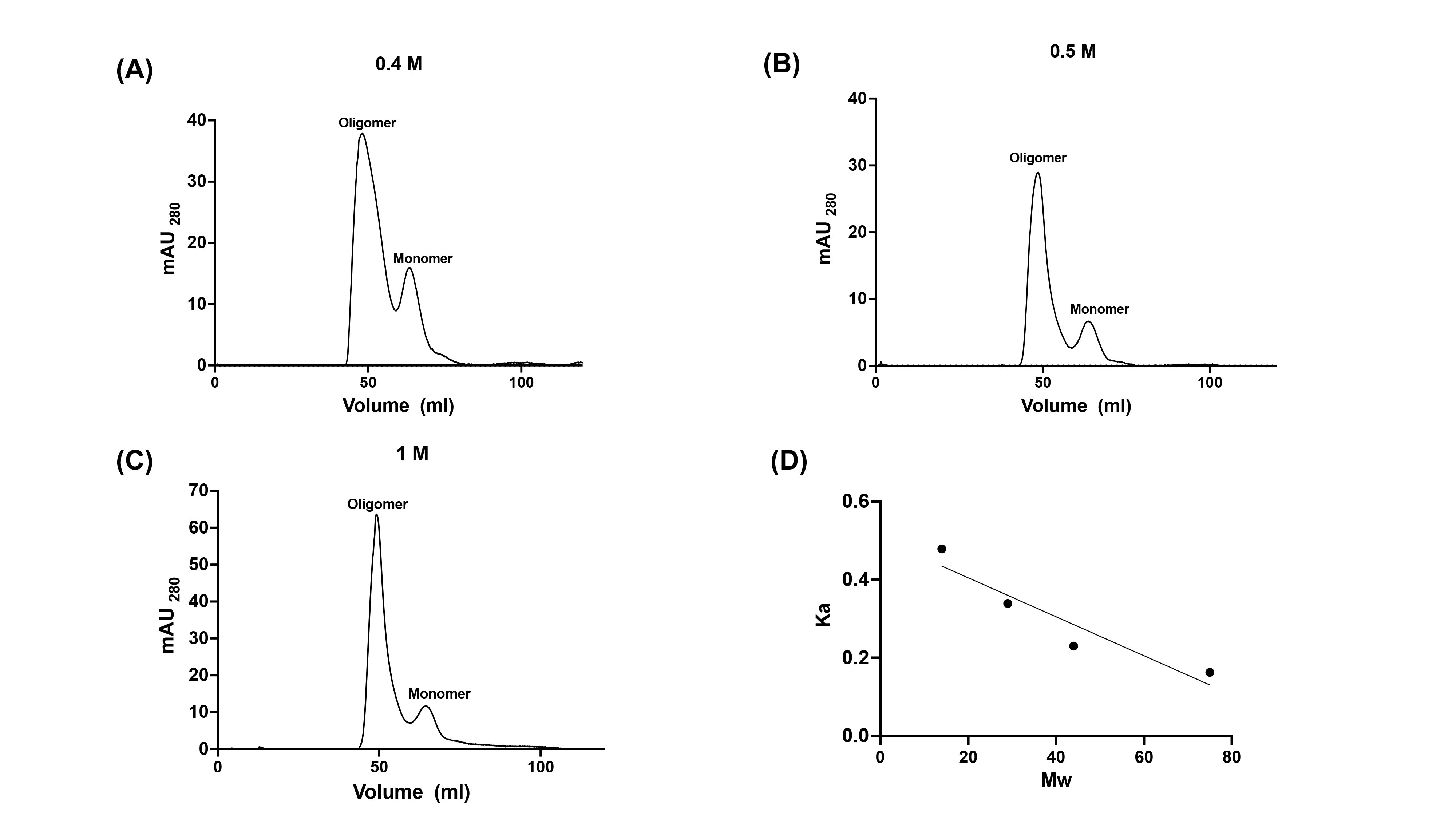

Supplement: Supplementary file 3 [file Image_3.jpeg]

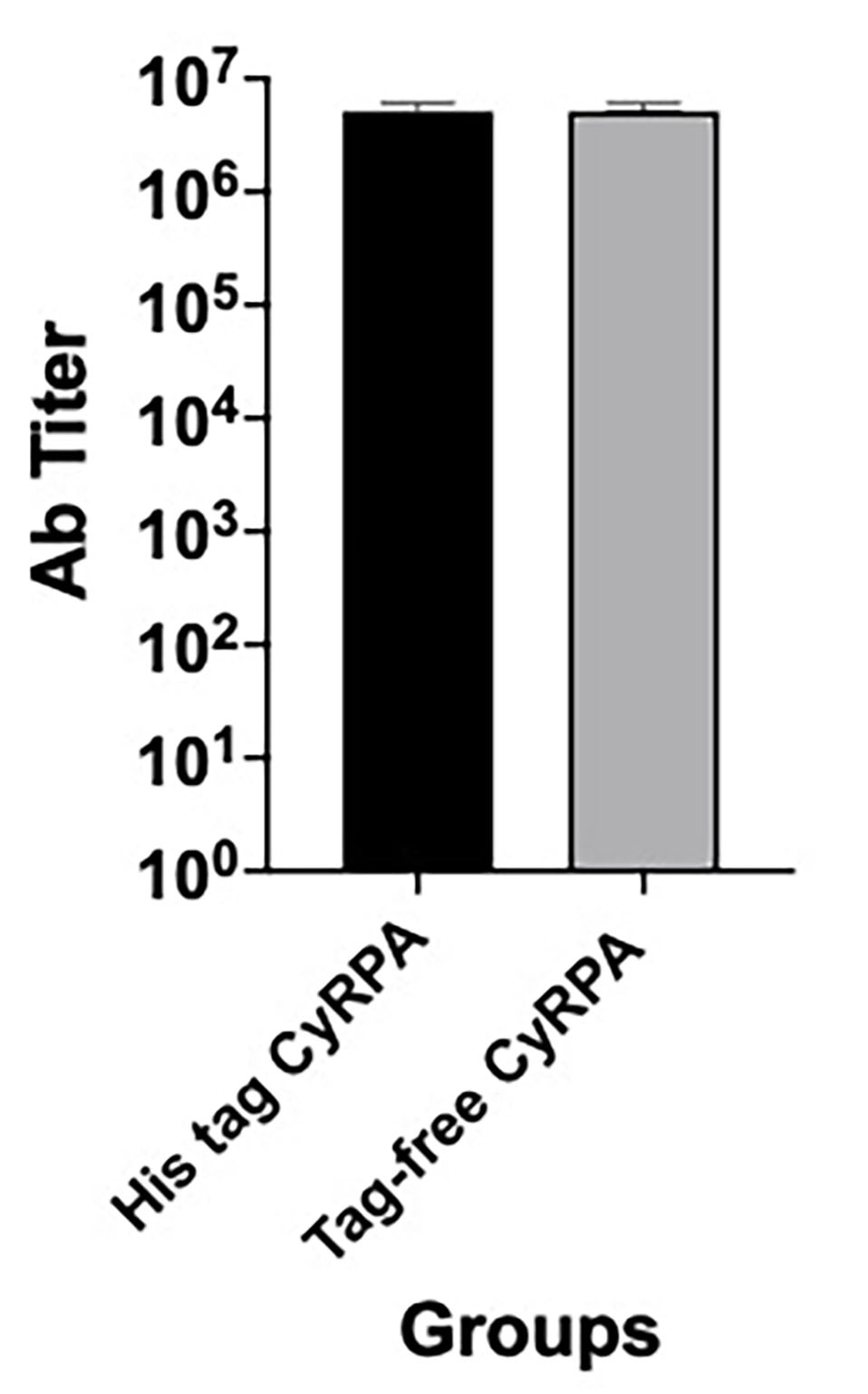

Supplement: Supplementary file 4 [file Image_4.jpeg]

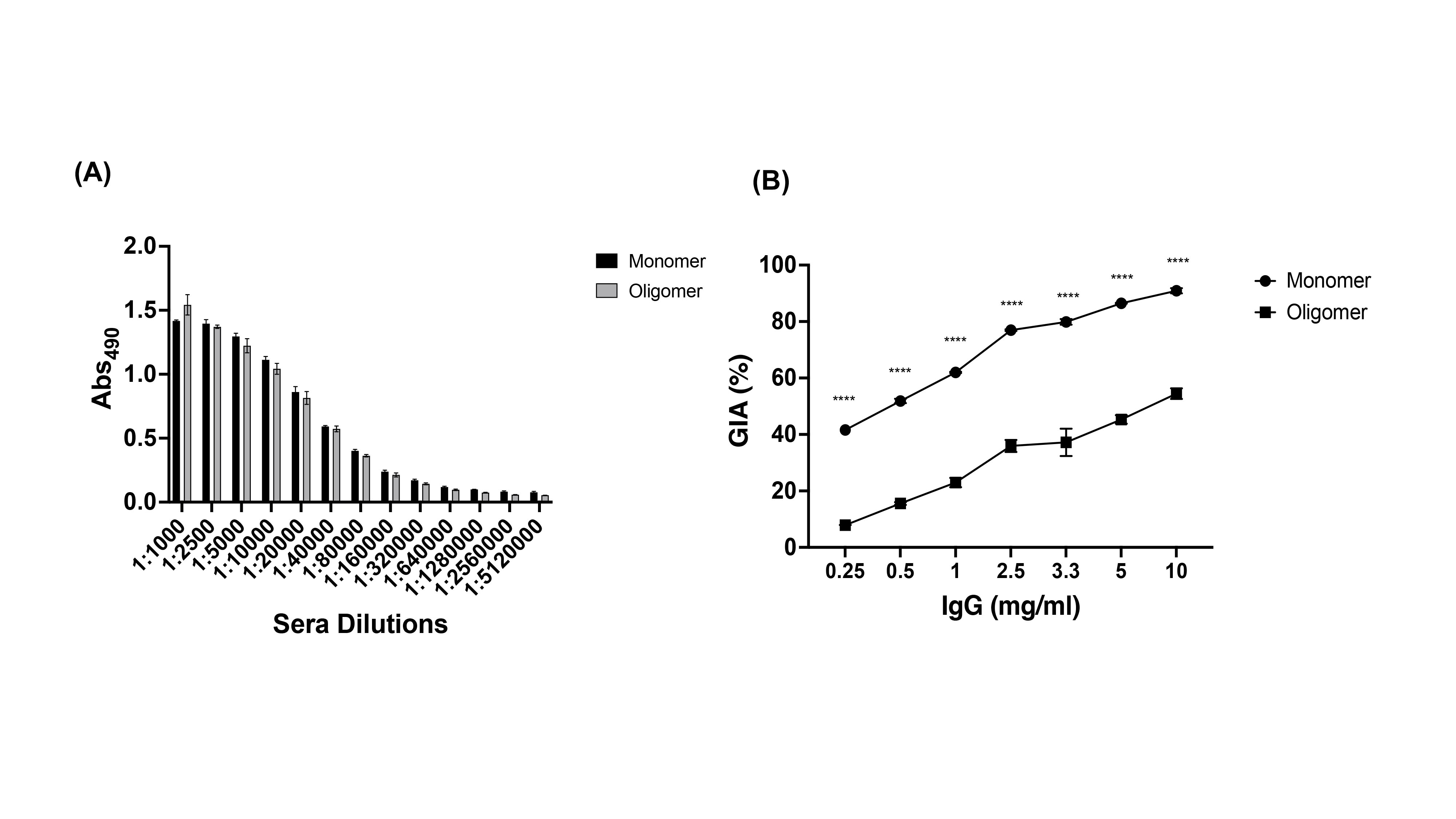

Supplement: Supplementary file 5 [file Image_5.jpeg]
