# Supplementary material for: Process development and preclinical evaluation of a major Plasmodium falciparum blood stage vaccine candidate, Cysteine-Rich Protective Antigen (CyRPA)
Source: Front Immunol. 2022 Sep 22;13:1005332. doi: 10.3389/fimmu.2022.1005332 (PMC9535676; doi:10.3389/fimmu.2022.1005332)
Supplement: Supplementary file 6 [file DataSheet_1.pdf]

Supplementary Information

Process Development and Preclinical Evaluation of a Major *Plasmodium falciparum* Blood Stage Vaccine Candidate, Cysteine-Rich Protective Antigen (CyRPA)

Anjali Somanathan[1], Syed Yusuf Mian[1#], Kritika Chaddha[1], Seemalata Uchoi[1], Praveen Bharti[2], Ravi Tandon[3], Deepak Gaur[1¶], Virander Singh Chauhan[4*]

[1]Laboratory of Malaria and Vaccine Research, School of Biotechnology, Jawaharlal Nehru University, New Delhi, India

[2]ICMR-National Institute for Research on Tribal Health (NIRTH), Jabalpur, Madhya Pradesh, India;

[3]Laboratory of AIDS research and Immunology, School of Biotechnology, Jawaharlal Nehru University, New Delhi, India

[4]Malaria Group, International Centre for Genetic Engineering and Biotechnology (ICGEB), New Delhi, India

[#]Current Address- Systems Immunology Laboratory, National Institute of Immunology, New Delhi, India

[¶]The authors dedicate this manuscript to the memory of beloved Prof. Deepak Gaur (Professor, School of Biotechnology, Jawaharlal Nehru University) who passed away with COVID-19

*Corresponding author

Address Correspondence to:

Prof. Virander Singh Chauhan

International Centre for Genetic Engineering and Biotechnology (ICGEB)

New Delhi, India 110067

Tel: +91-9811292058

E-mail: viranderschauhan@gmail.com

No. of supplementary tables- 2

No. of supplementary figures- 5

Table of Contents

1. **Tables**
2. **Figures Legends**

Supplementary Tables

Table S1. Mass Spectrometry Analysis of Purified Tag-free CyRPA. For mass spectrometric analysis (LC-MS) of 40 kDa CyRPA protein, the 0.3 M ion exchange elute was run on SDS-PAGE and the gel band was cut carefully for further investigation. The gel band was further subjected to alkylation and trypsin digestion to process the samples and analysed against PlasmoDB database. The master protein was identified to be CyRPA with an MS score of 10052 and 45 unique high-scoring peptides.

Master	Accession	Description	Score MS Amanda 2.0: MS Amanda 2.0	# Peptides	# PSMs	# Unique Peptides
**Master Protein**	**Q8IFM8**	**Cysteine-rich protective antigen OS=Plasmodium falciparum (isolate 3D7) OX=36329 GN=CyRPA PE=1 SV=1**	**10052.27**	**45**	**162**	**45**
**Confidence**	**Annotated Sequence**	**Modifications**	**Found in Sample: [S1] F1: Sample**	**# Protein Groups**	**# Proteins**	**# PSMs**
High	[M].ASHDKGETWGTK.[I]		High	1	1	1
High	[K].KDDILCMASHDKGETWGTK.[I]	1xCarbamidomethyl [C6]	High	1	1	3
High	[K].KVKDSWITLNDLFK.[E]		High	1	1	4
High	[C].LDDLKGEEDETHIYVQK.[K]		High	1	1	6
High	[C].LDDLKGEEDETHIYVQKK.[V]		High	1	1	6
High	[K].LGVQYFFLRPYISK.[N]		High	1	1	2
High	[D].LKGEEDETHIYVQK.[K]		High	1	1	1
High	[D].LKGEEDETHIYVQKK.[V]		High	1	1	1
High	[K].LLSSYVSLPLKIENR.[E]		High	1	1	4
High	[K].IVIKYDNYK.[L]		High	1	1	10
High	[K].NDLSFHFYVGDNINNVK.[N]		High	1	1	6
High	[S].NRDFLKDNK.[V]		High	1	1	3
High	[K].NVNFIECTHEK.[D]	1xCarbamidomethyl [C7]	High	1	1	5
High	[K].NVNFIECTHEKDLEFVCSNR.[D]	2xCarbamidomethyl [C7; C17]	High	1	1	4
High	[R].SINCDSRHVFIRTELSF.[I]	1xCarbamidomethyl [C4]	High	1	1	1
High	[C].SNRDFLK.[D]		High	1	1	1
High	[C].SNRDFLKDNK.[V]		High	1	1	5
High	[R].TELSFIK.[N]		High	1	1	2
High	[K].VKDSWITLNDLFK.[E]		High	1	1	2
High	[C].YGGTFVKIDENR.[T]		High	1	1	2
High	[K].GEEDETHIYVQKK.[V]		High	1	1	6
High	[K].GEEDETHIYVQK.[K]		High	1	1	4
High	[R].FYSNDGKEYNNSEITISDYILKDK.[L]		High	1	1	2
High	[R].FYSNDGKEYNNSEITISDYILK.[D]		High	1	1	4
High	[K].DDILCMASHDKGETWGTK.[I]	1xCarbamidomethyl [C5]	High	1	1	2
High	[R].DFLKDNK.[V]		High	1	1	18
High	[K].DKLLSSYVSLPLK.[I]		High	1	1	2
High	[K].DKLLSSYVSLPLKIENR.[E]		High	1	1	6
High	[K].DLEFVCSNR.[D]	1xCarbamidomethyl [C6]	High	1	1	5
High	[K].DLEFVCSNRDFLKDNK.[V]	1xCarbamidomethyl [C6]	High	1	1	2
High	[D].DLKGEEDETHIYVQK.[K]		High	1	1	3

High	[D].DLKGEEDETHIYVQKK.[V]		High	1	1	2
High	[R].DMFFIYKR.[E]		High	1	1	2
High	[K].YGNTTAGCYGGTFVK.[I]	1xCarbamidomethyl [C8]	High	1	1	2
High	[R].DMFFIYKR.[E]	1xOxidation [M2]	High	1	1	3
High	[K].DSWITLNDLFK.[E]		High	1	1	2
High	[C].EDEEFSNR.[K]		High	1	1	2
High	[R].ELYNICLDDLKGEEDETHIYVQK.[K]	1xCarbamidomethyl [C6]	High	1	1	2
High	[R].ELYNICLDDLKGEEDETHIYVQKK.[V]	1xCarbamidomethyl [C6]	High	1	1	2
High	[R].EYFLICGVSPYK.[F]	1xCarbamidomethyl [C6]	High	1	1	2
High	[R].EYFLICGVSPYKFK.[D]	1xCarbamidomethyl [C6]	High	1	1	2
High	[K].EYNNSEITISDYILK.[D]		High	1	1	4
High	[K].EYNNSEITISDYILKDK.[L]		High	1	1	6
High	[R].FYSNDGK.[E]		High	1	1	4
High	[K].DMTCHR.[F]	1xCarbamidomethyl [C4]	High	1	1	1
High	[K].YGNTTAGCYGGTFVKIDENR.[T]	1xCarbamidomethyl [C8]	High	1	1	3

Table S2. **Mass Spectrometry Analysis of 12 kDa CyRPA band.** To confirm the identity of the lower band, we performed mass spectrometric analysis (LC-MS) of 12 kDa protein by cutting the gel band from SDS-PAGE and subjecting it to alkylation and trypsin digestion followed by processing the samples and analysing against PlasmoDB database. The master protein in this case was also identified to be CyRPA with an MS score of 70 and 3 unique high-scoring peptides.

Master	Accession	Description	Score MS Amanda 2.0: MS Amanda 2.0	# Peptides	# PSMs	# Unique Peptides
**Master Protein**	**Q8IFM8**	**Cysteine-rich protective antigen OS=Plasmodium falciparum (isolate 3D7) OX=36329 GN=CyRPA PE=1 SV=1**	**70.81**	**3**	**5**	**3**
**Confidence**	**Annotated Sequence**	**Modifications**	**Found in Sample: [S2] F2: Sample**	**# Protein Groups**	**# Proteins**	**# PSMs**
High	[R].DFLKDNK.[V]		High	1	1	2
High	[R].FYSNDGKEYNN.[S]		High	1	1	2
High	[E].KDLEFVCSNR.[D]	1xCarbamidomethyl [C7]	High	1	1	1

Supplementary Figures Legends

Supplementary Figure S1. Expression of Tag-free CyRPA in GroEL-ES *E. coli* Cell Lines. Different *E.coli* strains- BL21 (DE3), C41 (DE3), Shuffle 26 (DE3) and Origami 2 (DE3) co-expressing a heterologous GroEL-ES chaperone were screened for soluble expression of tag-free CyRPA in the induced samples, in both the supernatant and pellet fractions as depicted in immunoblot which was probed with anti-CyRPA rabbit polyclonal sera. At small scale, all cell lines showed expression of protein in supernatant fractions, however, enrichment was not observed. * indicating the position of CyRPA. Abbreviations- S26: Shuffle 26; ORI2: Origami 2; I: Induced; S: Supernatant; P: Pellet.

Supplementary Figure S2. Immunoblot of Anion Exchange Elutes. Immunoblotting of 0.3 M, 0.4 M, 0.5 M and 1 M anion exchange elutes were probed with anti-CyRPA rabbit polyclonal sera. Alongwith 40 kDa band, a smaller band of 12 kDa was detected by the sera in 0.4 M, 0.5 M and 1 M elutes (observed in Figure 3B), indicating it to be a fragment of CyRPA. Higher order moieties of the protein are also detected in the elutes. * indicates position of CyRPA; arrowhead indicates position of 12 kDa band. Abbreviations- R: Reducing; NR: Non-reducing.

Supplementary Figure S3. Size Exclusion Chromatography Profiles of Anion Exchange Elutes. (A, B and C) Ion exchange elutes were loaded onto Superdex 75 (16/600) column instrument for monomeric or oligomeric state analysis by size exclusion chromatography (SEC). Two peaks each were observed in case of 0.4 M (A), 0.5 M (B) and 1 M (C) IEX elutes which indicates presence of higher order moieties or oligomers along with the monomeric fraction corresponding to the same elution volume in each SEC profile as calculated using the standard protein markers calibration curve depicted in (D). (D) Calibration curve was plotted

using the following protein standards: Conalbumin (75 kDa), Ovalbumin (44 kDa), Carbonic Anhydrase (29 kDa), Ribonuclease A (13.7 kDa). Elution profile of the protein molecular weight standards are shown. The curve was used to calculate the oligomeric and monomeric form of CyRPA. Abbreviations- Abs_{490}: absorbance at 490 nm; Mw- Molecular weight.

Supplementary Figure S4. Immunogenicity of Tag-free and (6-His) tagged CyRPA in Rabbits. NZW rabbits (n=1 per group) were immunized intramuscularly with tag-free CyRPA and (6-His) tagged CyRPA formulated with Freund's adjuvant (CFA/IFA). Enzyme-linked immunosorbent assay (ELISA) was performed with day 70 sera. Antibody titers were found to be as high as 51,20,000 for both immunized rabbits. ELISA cut-offs were calculated as the mean OD_{490} of pre-bleed sera plus three times standard deviation. The bars and error bars show mean and standard error values of 2 independent experiments conducted in duplicate.

Supplementary Figure S5. Immunogenicity and Parasite Neutralizing Efficacy of Monomeric Tag-free CyRPA vs Oligomeric Tag-free CyRPA Rabbit Antibodies. (A) NZW rabbits (n=1 per group) were immunized intramuscularly with tag-free CyRPA monomer and oligomer formulated with Freund's adjuvant (CFA/IFA). Enzyme-Linked Immunosorbent Assay (ELISA) was performed to calculate antibody titers in day 70 sera. Antibody titers were found to be as high as 25,60,000 for oligomer and 51,20,000 for monomer immunized rabbits. ELISA cut-offs were calculated as the mean OD_{490} of pre-bleed sera plus three times standard deviation. The bars and error bars show mean and standard error values of 2 independent experiments conducted in duplicate. (B). Purified rabbit total IgG against tag-free CyRPA monomer and oligomer were tested in the standard *in vitro* Growth Inhibitory Assay (GIA) against 3D7 clone in a one-cycle assay. Monomeric protein antibodies were more potent and showed parasite inhibition of ~90% at 10 mg/ml (**** $P \leq 0.0001$) compared to oligomeric protein antibodies which exhibited an inhibition of ~50% at 10 mg/ml. Data represents the

average of 2 independent experiments conducted in duplicate. The error bars represent the standard error between the 2 independent GIA experiments. The P values were calculated by two-way ANOVA with Bonferroni post-hoc testing.
